# Integrative analyses of genomic and metabolomic data reveal genetic mechanisms associated with carcass merit traits in beef cattle

**DOI:** 10.1038/s41598-022-06567-z

**Published:** 2022-03-01

**Authors:** Jiyuan Li, Yining Wang, Robert Mukiibi, Brian Karisa, Graham S. Plastow, Changxi Li

**Affiliations:** 1 Department of Agricultural, Food and Nutritional Science, University of Alberta, Edmonton, AB Canada; 2 Lacombe Research and Development Centre, Agriculture and Agri-Food Canada, Lacombe, AB Canada; 3 The Roslin Institute and Royal (Dick) School of Veterinary Studies, University of Edinburgh, Edinburgh, Scotland, UK; 4 Results Driven Agriculture Research, Edmonton, AB Canada

**Keywords:** Genome-wide association studies, Genetics, Animal breeding

## Abstract

Improvement of carcass merit traits is a priority for the beef industry. Discovering DNA variants and genes associated with variation in these traits and understanding biological functions/processes underlying their associations are of paramount importance for more effective genetic improvement of carcass merit traits in beef cattle. This study integrates 10,488,742 imputed whole genome DNA variants, 31 plasma metabolites, and animal phenotypes to identify genes and biological functions/processes that are associated with carcass merit traits including hot carcass weight (HCW), rib eye area (REA), average backfat thickness (AFAT), lean meat yield (LMY), and carcass marbling score (CMAR) in a population of 493 crossbred beef cattle. Regression analyses were performed to identify plasma metabolites associated with the carcass merit traits, and the results showed that 4 (3-hydroxybutyric acid, acetic acid, citric acid, and choline), 6 (creatinine, l-glutamine, succinic acid, pyruvic acid, l-lactic acid, and 3-hydroxybutyric acid), 4 (fumaric acid, methanol, d-glucose, and glycerol), 2 (l-lactic acid and creatinine), and 5 (succinic acid, fumaric acid, lysine, glycine, and choline) plasma metabolites were significantly associated with HCW, REA, AFAT, LMY, and CMAR (*P*-value < 0.1), respectively. Combining the results of metabolome-genome wide association studies using the 10,488,742 imputed SNPs, 103, 160, 83, 43, and 109 candidate genes were identified as significantly associated with HCW, REA, AFAT, LMY, and CMAR (*P*-value < 1 × 10^–5^), respectively. By applying functional enrichment analyses for candidate genes of each trait, 26, 24, 26, 24, and 28 significant cellular and molecular functions were predicted for HCW, REA, AFAT, LMY, and CMAR, respectively. Among the five topmost significantly enriched biological functions for carcass merit traits, molecular transport and small molecule biochemistry were two top biological functions associated with all carcass merit traits. Lipid metabolism was the most significant biological function for LMY and CMAR and it was also the second and fourth highest biological function for REA and HCW, respectively. Candidate genes and enriched biological functions identified by the integrative analyses of metabolites with phenotypic traits and DNA variants could help interpret the results of previous genome-wide association studies for carcass merit traits. Our integrative study also revealed additional potential novel genes associated with these economically important traits. Therefore, our study improves understanding of the molecular and biological functions/processes that influence carcass merit traits, which could help develop strategies to enhance genomic prediction of carcass merit traits with incorporation of metabolomic data. Similarly, this information could guide management practices, such as nutritional interventions, with the purpose of boosting specific carcass merit traits.

## Introduction

Carcass merit traits, including hot carcass weight (HCW), rib eye area (REA), average backfat thickness (AFAT), lean meat yield (LMY), and carcass marbling score (CMAR), are economically important traits in beef cattle since they directly influence the meat product yield and quality grade, and therefore profitability. For example, sufficient marbling is important for beef tenderness, juiciness and flavor, so the degree of marbling in beef is the primary factor determining quality grade of the meat. However, carcass merit traits are expressed late in life and the measurement of these traits for individual live animals is relatively expensive via ultrasound technologies. In many cases evaluation occurs post mortem, thereby eliminating breeding stock with superior breeding values for the traits. The development of genomic prediction provides an opportunity to assess genetic merit of animals as early as birth^1–4^ but there is still a need to improve the accuracy of genomic selection for carcass traits in beef cattle in order to achieve broader industry applications^3,5,6^. Detecting more candidate genes and functional or causal DNA variants through genome-wide association studies (GWAS) and understanding the biological background of the relationship between the genome and phenome could help improve the accuracy of genomic selection for complex traits including carcass merit traits^7–10^.

As more omics-based intermediate phenotypes, based on gene expression, protein and metabolite analysis, become available, integrating multi-omics data to further elucidate genetic influence of complex traits holds great promise^11–14^. Among the omics-based intermediate phenotypes, metabolites have been reported to be associated with carcass merit traits of livestock^15–17^ and their variation is influenced by genetic effects^18,19^. Therefore, we hypothesize that combining metabolomic data into GWAS of whole genome DNA variants could help detect key candidate genes and functional or causal DNA variants associated with carcass merit traits.

In this study, the data of 5 carcass merit traits (HCW, REA, AFAT, LMY, and CMAR) and 31 plasma metabolites were collected from a beef cattle population consisting of 493 crossbred bulls, heifers, and steers. Our objective was to identify significant single nucleotide polymorphisms (SNPs), candidate genes and biological functions associated with carcass merit traits through integration of carcass merit traits, plasma metabolites and whole genome sequence variants. Linear regression models were first used to identify metabolites associated with carcass merit traits. Metabolome-genome wide association studies (mGWAS) were then performed with 10,488,742 imputed whole genome SNPs to identify significant SNPs for the trait associated metabolites. Candidate genes were mapped based on significant SNPs and gene functional enrichment analyses were subsequently performed on candidate genes of each trait to predict biological functions/processes associated with carcass merit traits in beef cattle.

## Results

### Metabolites associated with carcass merit traits

The results of regression analyses showed 15 out of 31 analyzed metabolites were associated with one or more than one of the carcass merit traits (Table 1). At *P*-values less than 0.05, 3 (3-hydroxybutyric acid, acetic acid, and citric acid), 3 (creatinine, l-glutamine, and succinic acid), 1 (methanol), 1 (l-lactic acid), and 3 (succinic acid, lysine, and glycine) metabolites were identified as associated with HCW, REA, AFAT, LMY, and CMAR, respectively. However, some metabolites with a *P*-value from 0.05 to 0.1 explained more than 1% of the phenotypic variance of the associated carcass merit traits, thus, a relatively relaxed threshold of *P*-value < 0.1 was chosen to include more metabolites that may be potentially associated with carcass merit traits. For HCW, at *P*-values less than 0.1, 3-hydroxybutyric acid, acetic acid, citric acid, and choline were the associated metabolites, accounting for 1.92%, 1.69%, 1.48%, and 1.09% of the phenotypic variance in HCW, respectively. Creatinine, l-glutamine, succinic acid, pyruvic acid, l-lactic acid, and 3-hydroxybutyric acid were significantly associated with REA, and these six metabolites accounted for 1.87%, 1.79%, 1.60%, 1.56%, 1.04%, and 0.80% of phenotypic variance, respectively. AFAT was associated with fumaric acid, methanol, d-glucose, and glycerol, and these four metabolites explained 2.40%, 1.71%, 1.67%, and 1.39% of the phenotypic variance, respectively. l-lactic acid and creatinine were associated with LMY and accounted for 2.71% and 1.42% of phenotypic variance, respectively. Five metabolites, including succinic acid, fumaric acid, lysine, glycine, and choline, were associated with CMAR and each respectively accounted 1.76%, 1.36%, 1.22%, 1.20%, and 1.05% of the phenotypic variance in CMAR, respectively. Most of these metabolites were mainly associated with a single trait. However, a few metabolites were associated with more than one trait. For example, 3-hydroxybutyric acid was associated with both HCW and REA. l-lactic acid and creatinine were both associated with REA and LMY. Choline was associated with HCW and CMAR, and fumaric acid was associated with both AFAT and CMAR (Table 1). The additive genetic variance and heritability estimates of the 15 metabolites associated with the carcass merit traits were low to moderate (Table S1 in Supplementary file 1).

**Table 1:** A summary of metabolites associated with carcass merit traits in a multibreed population of beef cattle.

Trait^1^	Metabolite^2^	*P*-value^3^	b^4^	V_m_/V_P_ (%)^5^
HCW	3-Hydroxybutyric acid	1.01E−02	− 2.33E−01	1.92
	Acetic acid	1.56E−02	− 3.61E−02	1.69
	Citric acid	2.35E−02	− 1.10E−01	1.48
	Choline	5.31E−02	4.03E−02	1.09
REA	Creatinine	1.15E−02	2.50E−02	1.87
	l-glutamine	1.44E−02	5.51E−02	1.79
	Succinic acid	1.90E−02	− 3.46E−02	1.60
	Pyruvic acid	5.32E−02	1.87E−02	1.56
	l-lactic acid	5.94E−02	4.61E−04	1.04
	3-Hydroxybutyric acid	9.70E−02	− 2.47E−02	0.80
AFAT	Fumaric acid	4.05E−02	− 7.90E−02	2.40
	Methanol	4.81E−02	− 5.15E−03	1.71
	d-glucose	5.01E−02	− 8.64E−04	1.67
	Glycerol	7.29E−02	9.33E−04	1.39
LMY	l-lactic acid	1.21E−02	2.73E−04	2.71
	Creatinine	7.15E−02	7.41E−03	1.42
CMAR	Succinic acid	1.53E−02	− 2.18E−01	1.76
	Fumaric acid	7.83E−02	− 9.86E−01	1.36
	Lysine	4.36E−02	1.94E−01	1.22
	Glycine	4.55E−02	− 4.71E−02	1.20
	Choline	6.11E−02	− 3.80E−02	1.05

^1^*HCW* hot carcass weight in kg, *REA* rib eye area in cm^2^, *AFAT* average backfat thickness in mm, *LMY* lean meat yield in %, *CMAR* carcass marbling score from 100 (trace marbling) to 499 (more marbling).

^2^The unit of metabolite concentration is µM.

^3^The significance level of regression analysis is *P*-value < 0.1.

^4^*b* regression coefficient.

^5^*V*_*m*_*/V*_*P*_: the proportion of phenotypic variance of carcass merit traits explained by associated metabolites (%).

### Significant SNPs and candidate genes associated with metabolites

Genomic inflation factors for all association analyses ranged from 0.95 to 1.01 (Table S2 in Supplementary file 1), a value around 1 indicates that there is no population stratification, and the statistical models are well fitted. Summarized results of the mGWAS for the 15 metabolites (identified as associated with the carcass merit traits, *P*-value < 0.1) are presented in Table 2. Manhattan plots and QQ plots are provided in Figs. S3–S17 in Supplementary file 4. The average of phenotypic variance of the metabolites explained by a single SNP was 5.13% with a range of 3.57–10.95%. Details of significant SNPs for the 15 metabolites are provided in Supplementary file 2. The number of genes associated with the 15 metabolites varied from 3 (fumaric acid) to 53 (succinic acid) and a full list of candidate genes for each of the 15 metabolites is provided in Supplementary file 3.

**Table 2:** A summary of significant SNPs, the number of putative QTLs, and the number of candidate genes for metabolites associated with carcass merit traits in a multibreed population of beef cattle.

Metabolite^1^	*P*-value range^2^	$\beta$ range^3^	V_SNP_/V_P_ range (%)^4^	V_SNP_/V_P_ mean (%)^5^	No. of QTL^6^	No. of gene^7^
Acetic acid	2.46E−12 to 9.97E−06	− 259.54 to 181.91	4.01–10.95	5.05	31	49
Citric acid	1.47E−06 to 9.75E−06	− 29.80 to 37.62	3.57–4.95	4.05	15	15
Choline	4.94E−07 to 9.90E−06	− 89.13 to 84.25	3.85–5.43	4.61	13	23
d-glucose	6.82E−07 to 9.72E−06	− 226.21 to 257.62	3.70–5.67	4.33	18	23
Glycine	3.17E−06 to 9.54E−06	− 68.80 to 75.32	3.97–4.65	4.31	9	10
Glycerol	1.71E−07 to 9.76E−06	− 457.22 to 355.60	4.05–6.67	4.93	21	29
Fumaric acid	2.24E−07 to 9.83E−06	2.28 to 5.06	5.78–7.65	6.95	8	3
Lysine	9.11E−09 to 9.80E−06	− 17.77 to 20.56	3.88–7.13	4.82	15	20
l-lactic acid	2.24E−07 to 9.43E−06	− 1076.62 to 1261.24	3.74–5.95	4.58	16	21
Pyruvic acid	7.82E−08 to 9.99E−06	− 42.51 to 47.85	6.03–9.88	6.90	18	32
Succinic acid	3.32E−07 to 9.92E−06	− 30.10 to 25.93	3.94–6.19	5.01	26	53
3-Hydroxybutyric acid	8.55E−07 to 9.95E−06	11.24 to 19.78	4.00–5.13	4.46	12	19
Creatinine	1.64E−07 to 9.86E−06	− 26.59 to 31.04	3.78–6.31	4.67	17	22
l-glutamine	7.37E−07 to 9.90E−06	− 11.76 to 11.53	4.06–5.30	4.66	13	13
Methanol	3.15E−06 to 9.86E−06	− 32.86 to 45.32	4.00–4.82	4.30	15	28

^1^The unit of metabolite concentration is µM.

^2^The *P*-value range (minimum to maximum) of significant SNPs, the significance level is *P*-value < 1 × 10^–5^.

^3^$\beta$ range: the range of allele substitution effect of each significant SNP.

^4^V_SNP_/V_P_ range: the range metabolite phenotypic variance explained by each significant SNP (%).

^5^V_SNP_/V_P_ mean: the average of metabolite phenotypic variance explained by each significant SNP (%).

^6^No. of QTL: the number of putative QTLs identified for each metabolite.

^7^No. of gene: the number of candidate gene identified for each metabolite.

Through integrating the metabolite and carcass merit trait regression analyses and the mGWAS results, a total of 103, 160, 83, 43, and 109 candidate genes were found to be associated with HCW, REA, AFAT, LMY, and CMAR, respectively (Table 3). As for metabolites, some candidate genes identified through the mGWAS were associated with multiple carcass traits (Table S3 in Supplementary file 1 and Fig. S1 in Supplementary file 4). For instance, *CDH13* was associated with HCW, REA, AFAT, and CMAR, while 5 genes (*KMT5B*, *NDUFS8*, *ALDH3B1*, *CHKA*, and *TCIRG1*) were associated with HCW, REA, LMY, and CMAR.

**Table 3:** Metabolites and their candidate genes associated with carcass merit traits in a multibreed population of beef cattle.

Trait^1^	Metabolite^2^	Candidate gene
HCW	3-Hydroxybutyric acid	*RNASE1, RNASE6, RNASE4, ANG2, CDH13, TRAF3, AMN, CDC42BPB, PGM2, SULT1E1, CSN1S1, CSN2, HSTN, FRAS1, ANXA3, LOX, SRFBP1, NTRK2, COL12A1*
	Acetic acid	*PFN2, AGTR1, S100Z, CRHBP, AGGF1, LBH, YPEL5, ATAD2B, KLHL29, OR12K5, OR1B1, OR1L1, OR1L3, OR10W4, OR5B17, IZUMO2, MYH14, ZNF814, SNORA70, TMEM132E, HMGCLL1, GFRAL, TRAM2, TMEM14A, GSTA2, GPR139, UMOD, PDILT, ACSM5, ACSM2B, ACSM1, UQCRC2, PDZD9, MOSMO, VWA3A, IL21R, GTF3C1, KATNIP, ARMH3, HPS6, LDB1, PPRC1, SNORD22, DUSP5, SMC3, RBM20, KLRC1, APBB2, MAN2A1*
	Citric acid	*SERPINE3, INTS6, ZNF667, ZNF583, USP32, CA4, ZNHIT3, MYO19, TRAF3, AMN, CDC42BPB, EDEM1, ARL8B, KLHL31, SLC28A3*
	Choline	*HHAT, CDH8, PECAM1, MILR1, POLG2, DDX5, CEP95, ALDH3B1, NDUFS8, TCIRG1, CHKA, KMT5B, LRP5, PPP6R3, CPT1A, MRPL21, IGHMBP2, MRGPRF, CACNG2, IFT27, PVALB, BICD1, PERP*
REA	Creatinine	*ZBTB21, UMODL1, L3HYPDH, JKAMP, RTN1, PPP2R5E, MAML2, STOX2, ENPP6, IRF2, PRIMPOL, ACSL1, CENPU, RAB38, LUZP2, ALDH3B1, NDUFS8, TCIRG1, CHKA, KMT5B, LEPROT, DNAJC6*
	l-glutamine	*MYO16, UBE2E2, DDX56, NPC1L1, NUDCD3, CAMK2B, TRIM24, SVOPL, ATP6V0A4, PPP3CC, SORBS3, PDLIM2, CCAR2*
	Succinic acid	*GPR149, DHX36, ARHGEF26, PFN2, RNF13, HLTF, GYG1, AGTR1, ZIC1, ZIC4, SRSF5, SLC10A1, SMOC1, ACTR2, SPRED2, NDUFA8, MORN5, LHX6, RBM18, MRRF, PTGS1, OR1J2, OR1N2, OR1N1, OR1Q1, OR12K5, OR1B1, OR1L1, OR1L3, OR1AF3, OR1AF1, PDCL, RC3H2, ZBTB6, ZBTB26, RABGAP1, ZNF814, PLA2G2D1, PLA2G5, PLA2G2A, PLA2G2E, OTUD3, RNF186, TMCO4, LAP, DEFB13, RYR2, RBM47, NSUN7, APBB2, RAB28, NKX3-2, BOD1L1*
	Pyruvic acid	*SLC49A4, SEMA5B, HMGB1, CDX2, PDX1, GSX1, CHST8, KCTD15, NTN1, GAS7, KCNJ2, KCNJ16, DNER, RAB3C, PRL, GALR1, MBP, ZNF236, ZNF516, CNDP2, DIPK1C, C24H18orf63, CYB5A, STAB2, NT5DC3, HSP90B1, C5H12orf73, TDG, GLT8D2, PHF21B, NUP50, RIMS1*
	l-lactic acid	*PLSCR1, AQP9, NEDD4, PRTG, PYGO1, CUX2, NOS1, FBXO21, SPPL3, HNF1A, C17H12orf43, OASL, FOXN4, ACACB, TMEM171, FCHO2, CD247, POU2F1, MACF1, NPFFR2, SGCD*
	3-Hydroxybutyric acid	*RNASE1, RNASE6, RNASE4, ANG2, CDH13, TRAF3, AMN, CDC42BPB, PGM2, SULT1E1, CSN1S1, CSN2, HSTN, FRAS1, ANXA3, LOX, SRFBP1, NTRK2, COL12A1*
AFAT	Fumaric acid	*CDH13, SLC17A6, PPP2R2B*
	Methanol	*GRIK4, GRAMD1B, SCN3B, ZNF202, SMYD3, KIF26B, RGL1, CCDC92, DNAH10, PLA2G2A, PLA2G2E, OTUD3, MCTP2, XPC, TMEM43, CHCHD4, WNT7A, LUZP2, MARCHF1, PLAC8B, PLAC8A, COQ2, DENND4C, PLIN2, HAUS6, NEFM, NEFL, DOCK5*
	d-glucose	*TIAM1, KATNBL1, EMC7, CHRM5, AVEN, UBAC2, ST18, OR5M3, OR5M11, OR5AR1, INPP4B, PNKD, CATIP, SLC11A1, CTDSP1, VIL1, USP37, RARB, PGM5, TMEM252, WDR27, C9H6orf120, PHF10*
	Glycerol	*DGKG, SNX31, ANKRD46, BCO2, PTS, C15H11orf34, WIPF1, CPEB4, C20H5orf47, NSG2, CCDC88C, PPP4R3A, RAB23, BAG2, ZNF451, KCNK17, KCNK16, KIF6, STOX2, THRB, CDK14, TRBV15, LOX, SRFBP1, FSTL4, JADE2, SAR1B, SEC24A, TJP2*
LMY	l-lactic acid	*PLSCR1, AQP9, NEDD4, PRTG, PYGO1, CUX2, NOS1, FBXO21, SPPL3, HNF1A, C17H12orf43, OASL, FOXN4, ACACB, TMEM171, FCHO2, CD247, POU2F1, MACF1, NPFFR2, SGCD*
	Creatinine	*ZBTB21, UMODL1, L3HYPDH, JKAMP, RTN1, PPP2R5E, MAML2, STOX2, ENPP6, IRF2, PRIMPOL, ACSL1, CENPU, RAB38, LUZP2, ALDH3B1, NDUFS8, TCIRG1, CHKA, KMT5B, LEPROT, DNAJC6*
CMAR	Succinic acid	*GPR149, DHX36, ARHGEF26, PFN2, RNF13, HLTF, GYG1, AGTR1, ZIC1, ZIC4, SRSF5, SLC10A1, SMOC1, ACTR2, SPRED2, NDUFA8, MORN5, LHX6, RBM18, MRRF, PTGS1, OR1J2, OR1N2, OR1N1, OR1Q1, OR12K5, OR1B1, OR1L1, OR1L3, OR1AF3, OR1AF1, PDCL, RC3H2, ZBTB6, ZBTB26, RABGAP1, ZNF814, PLA2G2D1, PLA2G5, PLA2G2A, PLA2G2E, OTUD3, RNF186, TMCO4, LAP, DEFB13, RYR2, RBM47, NSUN7, APBB2, RAB28, NKX3-2, BOD1L1*
	Fumaric acid	*CDH13, SLC17A6, PPP2R2B*
	Lysine	*BTLA, ATG3, SLC35A5, CCDC80, CD200R1L, GTPBP8, NEPRO, BOC, SPICE1, SIDT1, FGF12, HS6ST3, FRMD5, MFHAS1, STYXL2, GPA33, DAB1, OR6C75, ITPR2, SSPN*
	Glycine	*AQP9, PHLDB1, TREH, DDX6, EIF5, MARK3, SEM1, PINX1, SOX7, C8H8orf74*
	Choline	*HHAT, CDH8, PECAM1, MILR1, POLG2, DDX5, CEP95, ALDH3B1, NDUFS8, TCIRG1, CHKA, KMT5B, LRP5, PPP6R3, CPT1A, MRPL21, IGHMBP2, MRGPRF, CACNG2, IFT27, PVALB, BICD1, PERP*

^1^*HCW* hot carcass weight in kg, *REA* rib eye area in cm^2^, *AFAT* average backfat thickness in mm, *LMY* lean meat yield in %, *CMAR* carcass marbling score from 100 (trace marbling) to 499 (more marbling).

^2^The unit of metabolite concentration is µM.

### Significantly enriched molecular functions and gene networks for carcass merit traits

**Figure 1:**
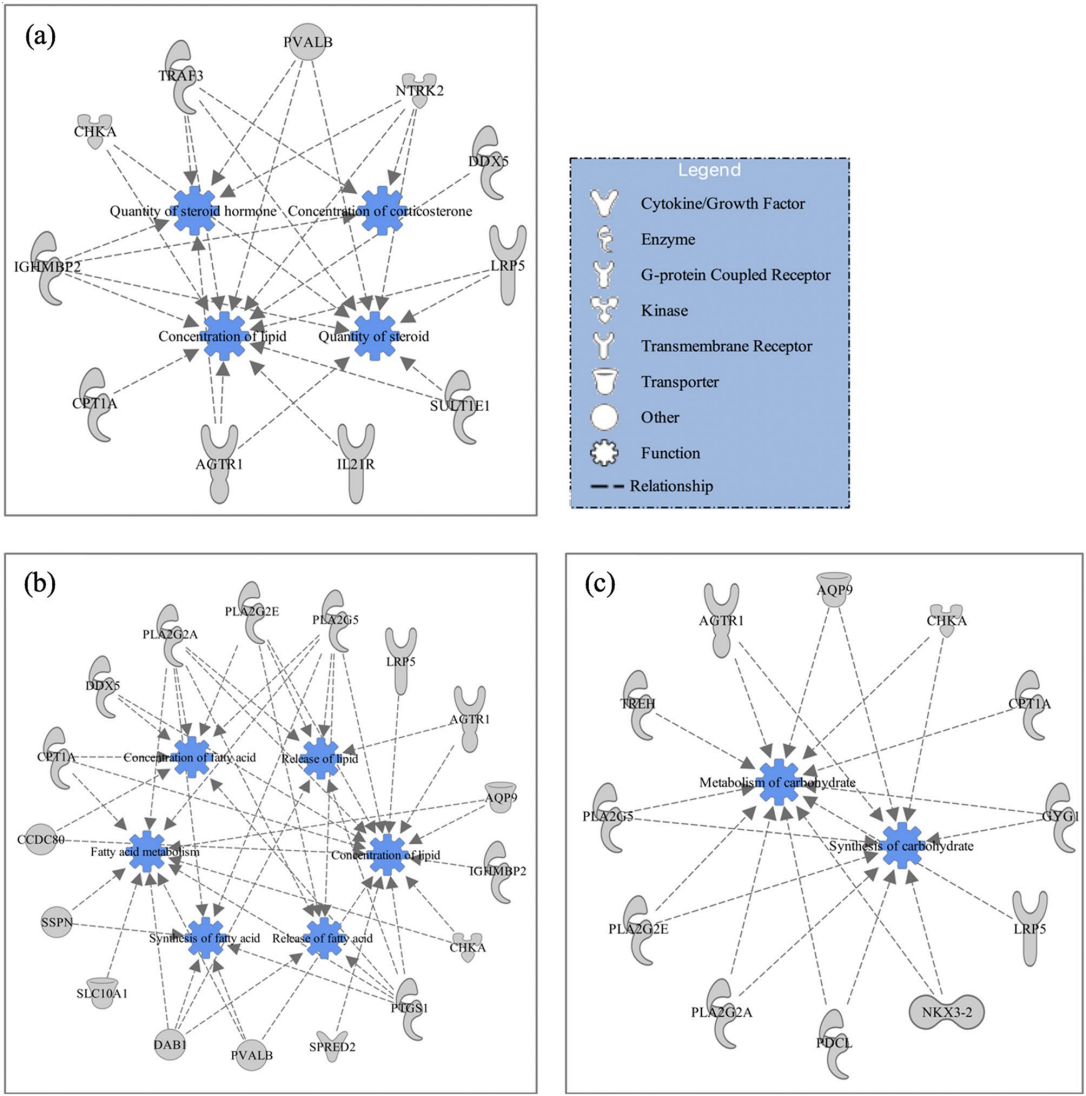
(**a**) gene network of molecular transport for hot carcass weight (HCW); (**b**) gene network of lipid metabolism for carcass marbling score (CMAR); (**c**) gene network of carbohydrate metabolism for carcass marbling score (CMAR).

Of the identified candidate genes for HCW, REA, AFAT, LMY, and CMAR, 99, 149, 78, 42, and 102 genes were respectively mapped to the IPA database for functional enrichment analyses. Briefly, 26, 24, 26, 24, and 28 cellular and molecular functions were identified as significantly (*P*-value < 0.05) associated with HCW, REA, AFAT, LMY, and CMAR, respectively (Tables S4–S8 in Supplementary file 1). Interestingly, 75% of the biological functions were commonly associated with all the five carcass merit traits in this study (Table S9 in Supplementary file 1 and Fig. S2 in Supplementary file 4). Some of the major functions common across the traits included molecular transport, small molecule biochemistry, lipid metabolism, cell-to-cell signaling and interaction, carbohydrate metabolism, cellular assembly and organization. Additionally, the five topmost significantly enriched biological functions for each trait and the candidate genes involved are presented in Table 4. Among the top five significant enriched functions, molecular transport and small molecule biochemistry were commonly associated with all carcass merit traits. Lipid metabolism was the most significant biological function for LMY and CMAR, and it was the second and fourth highest biological function for REA and HCW, respectively. Cell-to-cell signaling and interaction was one of the top significant biological functions associated with HCW, REA, AFAT, and CMAR. Carbohydrate metabolism was among the top significant biological functions associated with both HCW and CMAR. Further investigation within some of the biological functions revealed molecular/metabolic processes related to the carcass merit traits. For HCW, within the molecular transport function, 11 genes (*AGTR1*, *CHKA*, *CPT1A*, *DDX5*, *IGHMBP2*, *IL21R*, *LRP5*, *NTRK2*, *PVALB*, *SULT1E1*, and *TRAF3*) were involved in concentration of corticosterone and lipid, and quantity of steroid and steroid hormone (Fig. 1a). For CMAR, within the lipid metabolism function, 17 candidate genes (*AGTR1*, *AQP9*, *CCDC80*, *CHKA*, *CPT1A*, *DAB1*, *DDX5*, *IGHMBP2*, *LRP5*, *PLA2G2A*, *PLA2G2E*, *PLA2G5*, *PTGS1*, *PVALB*, *SLC10A1*, *SPRED2*, and *SSPN*) were involved in multiple metabolic processes related to fatty acid and lipid metabolism (Fig. 1b), such as synthesis of fatty acid and release of lipid. Additionally, within the carbohydrate metabolism function for CMAR, 12 candidate genes (*AGTR1*, *AQP9*, *CHKA*, *CPT1A*, *GYG1*, *LRP5*, *NKX3-2*, *PDCL*, *PLA2G2A*, *PLA2G2E*, *PLA2G5*, and *TREH*) were involved in carbohydrate metabolic processes such as carbohydrate biosynthesis (Fig. 1c). It is worth noting that 8 candidate genes (*AGTR1*, *AQP9*, *CHKA*, *CPT1A*, *LRP5*, *PLA2G2A*, *PLA2G2E*, and *PLA2G5*) associated with CMAR were involved in both lipid and carbohydrate metabolism.

**Table 4:** Five topmost significantly enriched biological functions for carcass merit traits, and genes involved in functions.

Trait^1^	Biological function	*P*-value range^2^	Genes involved in the biological function
HCW	Cell-to-cell signaling and interaction	8.91E−04 to 2.52E−02	*AGTR1, CACNG2, CDH13, CDH8, KLRC1, LOX, MAN2A1, NTRK2, PECAM1, PERP, PVALB, TRAF3, UMOD*
	Molecular transport	8.91E−04 to 2.52E−02	*AGTR1, CHKA, CPT1A, DDX5, HPS6, IGHMBP2, IL21R, KLRC1, LRP5, NTRK2, PECAM1, PVALB, SLC28A3, SULT1E1, TRAF3, UMOD*
	Small molecule biochemistry	8.91E−04 to 2.52E−02	*AGTR1, CHKA, CPT1A, DDX5, HMGCLL1, IGHMBP2, IL21R, KLRC1, LOX, LRP5, MAN2A1, NTRK2, PECAM1, PFN2, PGM2, PVALB, SLC28A3, SULT1E1, TRAF3, UMOD*
	Lipid metabolism	9.14E−04 to 2.52E−02	*AGTR1, CHKA, CPT1A, DDX5, HMGCLL1, IGHMBP2, IL21R, LRP5, NTRK2, PVALB, SULT1E1, TRAF3*
	Carbohydrate metabolism	1.35E−03 to 2.52E−02	*AGTR1, CPT1A, LRP5, MAN2A1, PGM2*
REA	Molecular transport	1.03E−06 to 6.53E−03	*ACACB, ACSL1, AGTR1, AMN, AQP9, ATP6V0A4, CAMK2B, CDX2, CHKA, CHST8, CSN2, DDX56, FRAS1, GALR1, GAS7, GSX1, HMGB1, HNF1A, IRF2, KCNJ16, KCNJ2, MBP, NEDD4, NOS1, NPC1L1, NTN1, NTRK2, NUP50, PFN2, PLA2G2A, PLA2G2E, PLA2G5, PLSCR1, PPP3CC, PRL, PTGS1, RAB3C, RIMS1, RYR2, SLC10A1, SORBS3, SPRED2, SRSF5, SULT1E1, TCIRG1, TRAF3, ZBTB21, ZIC1*
	Lipid metabolism	3.27E−05 to 6.53E−03	*ACACB, ACSL1, AGTR1, ALDH3B1, AQP9, CHKA, CHST8, CYB5A, ENPP6, GALR1, GAS7, HMGB1, HNF1A, IRF2, MBP, NOS1, NPC1L1, NTN1, NTRK2, PLA2G2A, PLA2G2E, PLA2G5, PLSCR1, PRL, PTGS1, RIMS1, SLC10A1, SPRED2, SULT1E1, TRAF3, ZBTB21*
	Small molecule biochemistry	3.27E−05 to 6.53E−03	*ACACB, ACSL1, AGTR1, ALDH3B1, AQP9, CHKA, CHST8, CNDP2, CYB5A, ENPP6, GALR1, GAS7, GSX1, HMGB1, HNF1A, IRF2, LOX, MBP, NOS1, NPC1L1, NTN1, NTRK2, PFN2, PLA2G2A, PLA2G2E, PLA2G5, PLSCR1, PPP3CC, PRL, PTGS1, RIMS1, RYR2, SLC10A1, SORBS3, SPRED2, STAB2, SULT1E1, TDG, TRAF3, ZBTB21*
	Cellular assembly and organization	9.97E−05 to 6.53E−03	*ACTR2, CAMK2B, CDC42BPB, CDH13, CUX2, DNAJC6, DNER, FCHO2, GAS7, HMGB1, KCNJ2, LOX, MBP, MYO16, NOS1, NTN1, NTRK2, NUDCD3, PFN2, PLSCR1, PPP3CC, PRL, RAB28, RAB38, RIMS1, RYR2*
	Cell-to-cell signaling and interaction	1.5E−04 to 6.53E−03	*AGTR1, CAMK2B, CD247, CDH13, CUX2, GALR1, HMGB1, HSP90B1, NOS1, NTN1, NTRK2, PFN2, PLA2G5, PPP3CC, PRL, RIMS1, SORBS3, TRAF3*
AFAT	Cell-to-cell signaling and interaction	1.63E−05 to 2.67E−02	*CDH13, CHRM5, GRIK4, LOX, MARCHF1, NEFL, NEFM, PNKD, PTS, RARB, SLC17A6, THRB, TIAM1, TJP2, WNT7A, XPC*
	Drug metabolism	1.63E−05 to 1.34E−02	*CHRM5, PNKD, PTS, SLC17A6*
	Molecular transport	1.63E−05 to 2.67E−02	*CDK14, CHCHD4, CHRM5, GRIK4, INPP4B, KCNK16, KCNK17, NEFM, PLA2G2A, PLA2G2E, PLIN2, PNKD, PPP2R2B, PTS, SCN3B, SEC24A, SLC11A1, SLC17A6, TJP2, WNT7A, XPC, ZNF202*
	Small molecule biochemistry	1.63E−05 to 2.34E−02	*BCO2, CHRM5, COQ2, DGKG, GRIK4, INPP4B, LOX, PLA2G2A, PLA2G2E, PLIN2, PNKD, PTS, SLC11A1, SLC17A6, THRB, WNT7A, XPC*
	Cellular assembly and organization	3.36E−05 to 2.67E−02	*CATIP, CDH13, CHCHD4, CHRM5, CPEB4, DGKG, DOCK5, KATNBL1, LOX, NEFL, NEFM, RAB23, RARB, TIAM1, TJP2, VIL1, WIPF1, WNT7A, XPC*
LMY	Lipid metabolism	1.18E−04 to 3.44E−02	*ACACB, ACSL1, ALDH3B1, AQP9, CHKA, ENPP6, HNF1A, NOS1, PLSCR1*
	Molecular transport	1.18E−04 to 3.8E−02	*ACACB, ACSL1, AQP9, CHKA, HNF1A, NEDD4, NOS1, NPFFR2, PLSCR1, TCIRG1, ZBTB21*
	Nucleic acid metabolism	1.18E−04 to 2.91E−02	*ACACB, ACSL1, AQP9, NOS1*
	Small molecule biochemistry	1.18E−04 to 3.44E−02	*ACACB, ACSL1, ALDH3B1, AQP9, CHKA, ENPP6, HNF1A, IRF2, NOS1, NPFFR2, PLSCR1, RAB38*
	Amino acid metabolism	1.84E−03 to 1.64E−02	*HNF1A, NOS1, RAB38*
CMAR	Lipid metabolism	1.57E−05 to 2.22E−02	*AGTR1, AQP9, CCDC80, CHKA, CPT1A, DAB1, DDX5, IGHMBP2, LRP5, PLA2G2A, PLA2G2E, PLA2G5, PTGS1, PVALB, SLC10A1, SPRED2, SSPN*
	Molecular transport	1.57E−05 to 2.36E−02	*AGTR1, AQP9, CCDC80, CHKA, CPT1A, DAB1, DDX5, IGHMBP2, LRP5, PECAM1, PLA2G2A, PLA2G2E, PLA2G5, PTGS1, PVALB, SLC10A1, SLC17A6, SPRED2*
	Small molecule biochemistry	1.57E−05 to 2.22E−02	*AGTR1, AQP9, CCDC80, CHKA, CPT1A, DAB1, DDX5, IGHMBP2, LRP5, PECAM1, PFN2, PLA2G2A, PLA2G2E, PLA2G5, PTGS1, PVALB, SLC10A1, SLC17A6, SPRED2, SSPN, TREH*
	Carbohydrate metabolism	4.1E−04 to 1.34E−02	*AGTR1, AQP9, CHKA, CPT1A, GYG1, LRP5, NKX3-2, PDCL, PLA2G2A, PLA2G2E, PLA2G5, TREH*
	Cell-to-cell signaling and interaction	2.47E−03 to 1.78E−02	*AGTR1, CACNG2, CDH13, CDH8, PECAM1, PERP, PFN2, PLA2G5, PVALB, SLC17A6*

^1^*HCW* hot carcass weight in kg, *REA* rib eye area in cm^2^, *AFAT* average backfat thickness in mm, *LMY* lean meat yield in %, *CMAR* carcass marbling score from 100 (trace marbling) to 499 (more marbling).

^2^The *P*-value range (minimum to maximum) of significant biological functions, the significance level is *P*-value < 0.05.

## Discussion

### Metabolomics to improve understanding on genetic influence of carcass merit traits

Studies have demonstrated metabolites as potential biomarkers for economically important traits in livestock species^15–17^. However, improving understanding of the biology involved is hampered by the limited knowledge of how these metabolites are associated with different economically important traits in different livestock species. Carcass merit traits are of fundamental interest to every beef producer and everyone involved in the beef industry. However, these traits are relatively expensive to measure using ultrasound technologies on individual live animals, which is a limitation for selection and improvement of these traits. Since blood metabolites are easily measurable/quantifiable even on live animals, we speculate that identification of genetic/biological associations between metabolite concentrations and beef cattle carcass merit traits could potentially enhance genetic prediction and selection for these traits in beef cattle. In addition, identification of blood metabolite biomarkers associated with carcass traits at an earlier stage would have a more practical application for genetic selection and for sorting animals into different finishing groups for more uniform carcass outputs. Therefore, we collected the blood samples at the start of the feedlot test instead of close to slaughter and examined the associations between 31 plasma metabolites and 5 carcass merit traits. We further explored the potential biological linkage between these metabolites and carcass merit traits. Our results showed that several metabolites were associated with the carcass merit traits studied. However, individual metabolites, despite being significantly associated with the trait, only accounted for 0.80–2.71% of the total phenotypic variance of carcass merit traits. This relatively small percentage of phenotypic variance reflected the complex nature of these traits, which we believe are regulated by multiple metabolic pathways involving many metabolites with each having only a small contribution/effect. It is also possible that due to the limited number of metabolites we profiled in the current study, we were not able to identify those metabolites with major influences on the traits studied. Additionally, this study used a more relaxed threshold (*P*-value < 0.1) to identify metabolites potentially associated with carcass merit traits, therefore, validation in independent beef cattle populations or further studies considering a wider range of metabolites is warranted. It is also worthwhile to analyze metabolites on samples collected at different developmental stages to see whether and how the associations between the metabolites and the carcass traits may change. Furthermore, we observed that a majority of the significant metabolites were only associated with one trait. However, some metabolites in the current study were associated with two traits, indicating potential biological relationships between these traits. For example, in this study, we observed that 3-hydroxybutyric acid was associated with both HCW and REA, and beef cattle with high HCW and REA had lower concentration of 3-hydroxybutyric acid, indicating that animals with high HCW and REA may have better carbohydrate metabolism. Additionally, 3-hydroxybutyrate is the main representative of ketone bodies and one important function of ketone bodies is to provide acetoacetyl-CoA and acetyl-CoA for the synthesis of cholesterol, fatty acids, and complex lipids^20^. Thus, a lower concentration of 3-hydroxybutyric acid may lead to reduced lipid synthesis in animals with high HCW and REA. Interestingly, creatinine, the final catabolite of muscle energy metabolism^21^, was positively associated with both REA and LMY in the current study (Table 1), and these two carcass merit traits measure muscle development and the proportion of lean meat in a carcass respectively. In line with our results, Hanset et al.^22^ previously observed higher concentrations of plasma creatinine in double muscled bulls as compared to conventional or normal muscled bulls, and Patel et al.^23^ proposed creatinine in serum as a promising biomarker for human muscle mass. These previous studies and the results from our study demonstrate that creatinine is a potential indicator trait or biomarker for muscle related traits in beef cattle. Finally, the heritability of metabolites estimated in this study could be used as reference information. Large standard errors for these estimates were observed due to the sample size used and future research utilising a larger sample size is warranted.

### Candidate genes, enriched molecular functions and gene networks for carcass merit traits

Generally, identification of SNPs and genes associated with carcass merit traits mainly relies on association studies between DNA variants and the traits. For example, Wang et al.^24^ performed GWAS based on 7.8 million imputed whole genome sequence variants for carcass merit traits using Canadian beef cattle and they identified hundreds of candidate genes associated with carcass merit traits. However, the knowledge about the underlying biological background behind these associations is relatively limited. We assume that metabolites, which are an intermediate phenotype lying between genome and carcass merit traits, could provide additional insight into the associations. In the current study, the candidate genes identified through incorporating metabolites showed relatively good consistency with the previous study^24^ (Table S10 in Supplementary file 1). Briefly, we found that 34.95%, 28.13%, 27.71%, 41.86%, and 22.94% of the candidate genes identified in this study overlapped with those from Wang et al.^24^ for HCW, REA, AFAT, LMY, and CMAR, respectively. Of note, some of the candidate genes were also reported in different cattle breeds or populations in other studies. For example, *ST18* was associated with AFAT in the current study and it was associated with the metabolite d-glucose. Medeiros de Oliveira Silva et al.^25^ also identified *ST18* as candidate gene for backfat through GWAS in a Nelore cattle population. Additionally, by integrating metabolomic data, this study added more information to some previously identified associations between genes and carcass merit traits. For example, Wang et al.^24^ reported that *UMODL1*, *L3HYPDH*, *JKAMP,* and *LUZP2* were candidate genes associated with REA and LMY, but the potential mechanism of how these genes could influence the two traits remained unclear. Our study showed that these same genes (*UMODL1*, *L3HYPDH*, *JKAMP*, and *LUZP2*) were associated with the concentration of creatinine which is a metabolite associated with REA and LMY. These results indicated that these genes may be associated with the synthesis or degradation of creatinine in animals and thereby influence the related traits. Similarly, *HLTF*, *GYG1*, *RYR2*, *RBM47*, and *APBB2* were reported to be associated with REA and CMAR by Wang et al.^24^. Our results showed these genes were associated with succinic acid which was negatively associated with both REA and CMAR. Both examples represent one of situations that genes may influence different traits by regulating the same metabolites, and the mechanism of how these genetic variants affect the concentration of metabolites still needs more studies. According to our results, we would like to highlight that some genes could affect the same carcass merit traits by influencing different metabolites. For instance, *AMN* was associated with both 3-hydroxybutyric acid and citric acid, and both metabolites were identified as associated with HCW. Therefore, information obtained via analyzing metabolites could improve the understanding of genetic effects on these phenotypes. In our companion paper by Li et al.^26^, similar findings were also observed. These two studies indicate that metabolites play important roles in the variation of both feed efficiency and carcass merit traits. Integration of metabolomic and genomic data could help identify functional or causal SNPs or genes, and interpret the biological meaning of the candidate genes identified in GWAS. In addition, these two studies investigated the associations between different omics levels, which could shed light on the interrelationship between different omics layers and potential molecular mechanisms underlying these traits. Therefore, our findings have broadened our knowledge on the genetic and molecular mechanisms of these traits. Based on what we have learned from these two studies, we recommend applying such multi-omics analyses to study other important traits in beef cattle.

In addition to adding more information to known associations, incorporating metabolomic data can help us identify additional novel associations as metabolites represent a level close to the final phenotypes (i.e., carcass merit traits). In the current study, some additional candidate genes were reported to be associated with carcass merit traits. Therefore, we expect that including the candidate gene SNPs in the DNA marker panel or increasing the weight applied to such SNPs could either improve accuracy of genomic prediction of the traits or decrease the DNA marker density used in genomic prediction while retaining accuracy. A preliminary attempt of this latter option was done by Melzer et al.^27^ for the prediction of three traditional milk traits in dairy cows. Melzer et al.^27^ applied regression methods to identify important milk metabolites and then those SNPs with significant genetic effects on important metabolites were identified and used to predict milk traits. Compared with the classical approach that uses all SNPs (40,317) in prediction, this metabolite approach could achieve similar prediction precision with less than 1% of the total amount of SNPs. Fontanesi^28^ suggested integration of metabolomic data would be useful if the heritability of a trait is low, if a trait is hard to be precisely and directly measured on the animals, or if the prediction accuracy was limited by the small number of phenotyped animals in the training populations. Since carcass merit traits are expressed at later stages of animal production and are usually measured by sacrificing potential breeding stock, these traits are more suitable for DNA marker based genomic prediction, and incorporating metabolomic data into the genomic prediction has the potential to enhance the genomic prediction accuracy. In addition to metabolites, the information carried by other omics data, such as RNA and protein, also helps to prioritize SNPs associated with complex traits, and can further contribute to improving genomic prediction accuracy of these traits. For example, Fang et al^29^ applied an extended genomic best linear unbiased prediction (GBLUP) model called genomic feature BLUP (GFBLUP) that included a separate random effect for the joint action of SNPs within genomic features which were obtained from RNA differential expression analyses. Compared to GBLUP, the accuracy of genomic prediction for mastitis and milk production traits with GFBLUP was marginally improved (3.2 to 3.9%) in within-breed prediction but significantly increased (164.4%) in across-breed prediction. Theoretically, the genomic features could be defined from various sources of biological knowledge (e.g., metabolomics) and the GFBLUP model could be applied to other complex traits. Therefore, it would be of interest to investigate the accuracy of prediction for carcass merit traits with and without utilizing multi-omics data.

In order to further investigate biological functions associated with carcass merit traits, five topmost significant biological functions associated with each trait were identified in the current study. These top five biological functions showed substantial overlap with the top five biological functions identified by previous studies^24,30,31^, which indicated those overlapping top biological functions potentially have important biological meaning for the carcass merit traits in beef cattle. Since our carcass data were collected from animals that were finished for meat production, genes involved in these overlapping top biological functions, such as lipid metabolism and carbohydrate metabolism, likely play a more important role in determining the carcass merit traits. Therefore, the identification of top and other enriched biological functions and their corresponding genes will not only improve our understanding of the underlying biology but also help prioritize candidate genes and related putative causal SNPs for future studies. Additionally, construction of gene networks for biological functions could help us elucidate complicated connections among genes and disentangle the potential relationships among genes, biological functions and phenotypes. For example, molecular transport was identified as a top enriched biological function associated with all carcass merit traits and its network of HCW as an example showed that some of the associated genes were involved in concentration of lipid and corticosterone, and quantity of steroid and steroid hormones (Fig. 1a). In beef cattle production, more than 30 commercially-available steroid hormone implants are marketed in the U.S. and the effects of steroid hormone implants on improving carcass leanness, increasing average daily gain, and altering dry matter intake has been reviewed by Smith and Johnson^32^. Thus, those genes linked to the functions of steroid and steroid hormones in the network may consequently influence final muscle mass in the carcass. For those genes involved in the concentration of lipid, they may influence fats in the carcass by regulating breakdown or storage of fats. Additionally, we would like to highlight the network of lipid metabolism for CMAR (Fig. 1b) because lipid metabolism was the most significant biological function associated with this trait. In this network, some genes were involved in fatty acid metabolism including fatty acid synthesis, release and concentration. The phenotypic and genetic correlations between fatty acid composition and marbling have been reported in different beef cattle populations^33–36^. Our results provide further insight into the potential molecular and genetic background accounting for genetic correlations between marbling and fatty acid composition in beef cattle, further indicating that the selection for fatty acid composition or concentration could influence marbling in beef cattle as previously proposed^36^.

## Conclusion

In this study, genomic, metabolomic, and phenotypic data were integrated to investigate biological functions/processes related to carcass merit traits in beef cattle. Plasma metabolites associated with HCW, REA, AFAT, LMY, and CMAR were identified and individual metabolites were found to account for a small proportion of the total phenotypic variance of the carcass merit traits. Several candidate genes as associated with carcass merit traits were identified through mGWAS along with regression analyses. These genes are involved in multiple biological functions that are related to the associated carcass merit traits. Additionally, the results of our integrative analyses could help interpret previous results from DNA marker based GWAS of the carcass merit traits and revealed functional genes associated with these economically important traits. Therefore, our integrative study has provided insights into relationships between genes, metabolites and carcass merit traits, which could potentially lead to improvement of genomic prediction accuracy via incorporating metabolomic related data.

## Material and methods

### Animal population, metabolomic and phenotypic data collection

All animals in this study were cared for according to the guidelines of the Canadian Council on Animal Care (2009) and the experimental procedures were approved by the University of Alberta Animal Care and Use Committee (AUP00000777). In total, 493 bulls (n = 93), heifers (n = 125) or steers (n = 275) from different herds including Charolais (n = 73), Hereford-Angus crosses (n = 191), and Beefbooster composite breed (predominantly Charolais-based, n = 229) were used. Among these animals, 277 animals from two herds had implants, while 216 animals from the other three herds had no implant. The effect of the factor of implant was examined using statistical analysis, and its effect has been captured under the herd variable through subsequent phenotypic value adjustment. Animals were born between 2002 to 2011 and initially measured for feed intake using the GrowSafe system (GrowSafe Systems Ltd., Airdrie, Alberta, Canada) at the feedlot test station under multiple projects, which were described previously^37–40^. The animals from the same herd of a particular year were tested in the same feedlot and diet. Blood samples were collected from all animals by jugular venipuncture in the early morning on the first day of feedlot feeding test and immediately frozen at − 80 °C for storage. Plasma metabolites were quantified using nuclear magnetic resonance (NMR) spectroscopy as described by Li et al.^18^. Briefly, blood samples were thawed at room temperature and centrifuged at 10,000 rpm for 10 min to separate the plasma. Plasma was then filtered through 3 kDa molecular weight cut-off filters (Merck Millipore Ltd. 3KDA filter tubes; Darmstadt, Germany) to exclude macromolecules, including lipids and proteins. After filtration samples with a low volume were diluted with high-performance liquid chromatography (HPLC) water to 570 μl to ensure an adequate volume for NMR acquisition. Samples were further prepared in a 5 mm NMR tube (New Era Enterprises Inc., NJ, USA) that contained a total of 700 μl including 570 μl filtered serum, 60 μl DSS and 70 μl D2O. Spectra were acquired on a 500 MHz VNMRS spectrometer equipped with a 5 mm cold probe (Agilent Technologies, CA, USA). Metabolites were identified and quantified with a targeted profiling approach using the Profiler and Library Manager modules in the same software which contained 304 total metabolites as references. Each spectrum was reviewed by a separate analyst and a final review was performed on all of the spectra before exporting concentration results. Concentration measurements were adjusted to report metabolite concentrations (µM). In total, 33 metabolites were initially identified and quantified using NMR. However, two of them were excluded due to missing values, resulting in 31 metabolites for further analyses.

In order to collect carcass data, animals were then slaughtered after the feedlot tests at either a commercial plant or the Lacombe Research and Development Centre (LRDC) abattoir when a majority of them reached > 8 mm backfat as predicted from ultrasound measurement. The processes of carcass data collection were previously described^39,41–45^. In summary, hot carcass weight (HCW) in kg was obtained by summing up the weight of each side of the carcass that was split during dressing, about 45 min post-mortem. Average backfat thickness (AFAT) in mm, rib eye area (REA) in square centimeters, and carcass marbling score (CMAR) at the grading site between the 12th and 13th ribs was assessed by trained personnel. Carcass marbling score was measured as a continuous variable from 100 (trace marbling or less) to 499 (abundant or more marbling) to reflect the amount of fat deposit interspersed between the muscle fibers (i.e., intramuscular fat) of the longissimus thoracis. Lean meat yield (LMY) was calculated as LMY, % = 57.96 + (0.202 × REA, cm^2^) − (0.027 × HCW, kg) − (0.703 × AFAT, mm) as an estimate of saleable meat in the carcass^37^.

### Animal genotyping, SNP imputation and quality control

DNA was extracted from the blood samples using DNeasy Blood & Tissue Kit (QIAGEN, Ontario, Canada) and then genotyped using the Illumina BovineSNP50 v2 BeadChip (Illumina Inc., CA, USA). For the SNP imputation, a step-wise procedure was applied as described in our previous studies^24,40^ using Beagle 5.1 software^46^. Briefly, we first imputed from the 50 K SNPs to the AffyHD panel of 444,558 SNPs with 4,247 animals of mixed beef breeds in the reference population. We then imputed from the imputed AffyHD panel to the whole genome sequence variants with the reference population of 3,093 Bos taurus animals from the 1000 Bull Genomes Project^47^ (run 7). Finally, 53,258,178 variants (SNPs and indels) on 29 autosomes were obtained after the imputation with average imputation accuracy of 0.97 across the DNA variants with a standard deviation (SD) of 0.08, which was assessed through a five-fold cross-validation as described in our previous studies^24,40^. For each SNP, post-imputation quality control was then performed to filter out the imputed variant genotypes if one of the following conditions was met: (1): SNPs on 29 autosomes that had an imputation accuracy < 0.95; (2): minor allele frequency < 0.05; (3) SNPs failed to pass the Hardy–Weinberg equilibrium test (P-value < 0.0001). A total of 10,488,742 SNPs remained for subsequent analyses after the quality control.

### Regression analyses between carcass merit traits and metabolites and metabolome-genome wide association studies

Phenotypic values of carcass merit traits and metabolites were adjusted for factors including animal type (bull, heifer, steer), birth year, herd, feedlot pen, animal age at slaughter, and breeding composition using linear regression models. The breed composition of each animal was predicted based on their 50 K SNPs using ADMIXTURE software to account for population stratification^48,49^. The value of *K* = 6 was chosen because it had the smallest cross-validation error and yielded the most accurate breed composition prediction based on prior knowledge of breed composition on a subset of animals. Residuals of metabolomic and phenotypic data beyond 3 standard deviations from the mean of residuals were considered as outliers and excluded from further analyses. The descriptive statistics of phenotypic data on carcass merit traits and metabolites are shown in Table S11 in Supplementary file 1. In order to determine relationships between carcass merit traits and metabolites, regression analyses were conducted between the adjusted carcass merit traits and the 31 adjusted metabolites using R statistical software. A carcass merit trait and a metabolite were considered to be significantly associated when the regression analyses have a *P*-value < 0.1.

For the metabolome-genome wide association studies (mGWAS), the adjusted values of metabolites that were significantly associated with the carcass merit traits were the response variable in the single SNP-based mixed linear model association (*mlma*) as implemented in GCTA software^50^. The linear mixed model can be described as follows:

$${y}_{ij}=\mu +{b}_{j}{x}_{ij}+{a}_{ij}+{e}_{ij}$$

where ${y}_{ij}$ is the adjusted metabolite value of the $i$ th animal with the $j$ th SNP (i.e. the $ij$ th animal), ${b}_{j}$ is the allele substitution effect of the $j$ th SNP, ${x}_{ij}$ is the $j$ th SNP genotype of animal $i$ coded as 0, 1, 2 for genotypes ${A}_{1}{A}_{1}$, ${A}_{1}{A}_{2}$, and ${A}_{2}{A}_{2}$, respectively, ${a}_{ij}$ is the additive polygenic effect of the $ij$ th animal $\sim N(0,{\varvec{G}}{\sigma }_{a}^{2})$, and ${e}_{ij}$ is the random residual effect $\sim \boldsymbol{ }N(0,\boldsymbol{ }{\varvec{I}}{\sigma }_{e}^{2}$). The genomic relationship matrix ${\varvec{G}}$ was derived based on total filtered SNP markers (i.e. 10,488,742 SNPs) as described by Yang et al.^51^, which is essentially the same as the second VanRaden’s formulation^52^. The SNP allele substitution effect was estimated and the significance test of the SNP allele substitution effect was conducted via a generalized least square F-test as implemented in the GCTA package. The SNPs with *P*-value < 1 × 10^–5^ were considered to be significantly associated with the metabolite according to the recommendation of The Wellcome Trust Case Control Consortium^53^. The phenotypic variance of the metabolite explained by each significant SNP was calculated by $\frac{2pq{\beta }^{2}}{{S}^{2}}*100\mathrm{\%}$, where $p$ and $q$ denote the SNP allele frequency of ${A}_{1}$ and ${A}_{2}$, respectively; $\beta$ is the SNP allele substitution effect; $2pq{\beta }^{2}$ is the additive variance of the SNP, and ${S}^{2}$ is the phenotypic variance of the metabolite.

### Identification of candidate gene and functional enrichment analyses for carcass merit traits

A 140-kbp window (70-kbp upstream and 70-kbp downstream) of each significant SNP was used to map candidate genes based on ARS-UCD 1.2 bovine genome assembly from the Ensembl BioMart database (accessed in February 2021). The 70-kbp was the chromosomal length within which a high linkage disequilibrium phase correlation (${r}^{2}$> 0.77) was maintained across a sample of Canadian beef cattle breeds^54^.

The Entrez gene IDs were used as gene identifiers and small nucleolar RNA and microRNA were excluded from gene functional enrichment analyses. Bovine genes were changed to known human orthologous genes from Ensembl, whereas for those genes without human orthologs their bovine gene IDs were maintained in the gene list. Then candidate genes of all metabolites associated with the carcass merit traits (HCW, REA, AFAT, LMY or CMAR) as identified in the regression analyses between carcass merit traits and metabolites were combined and imported into the Ingenuity Pathway Analysis software (accessed in February 2021) (IPA; www.Ingenuity.com). Significantly enriched molecular and cellular biological functions and gene networks (*P*-value < 0.05) for each carcass merit trait were inferred and gene-sub-biological function/process interactions within the most significant molecular and cellular functions were predicted in the IPA.

## Supplementary Information

Supplementary Information 1.

Supplementary Information 2.

Supplementary Information 3.

Supplementary Information 4.

## Data Availability

The dataset supporting the results of this article are included within the article and its supplementary files. Whole genome sequence datasets generated and/or analyzed during the current study for imputation are available from the NCBI SRA database under BioProjects PRJNA176557 and PRJNA256210. The original genotype and phenotype data sets are available for non-commercial purposes from GP or CL following the execution of a materials transfer agreement.
