# Supplementary material for: Integrative analyses of genomic and metabolomic data reveal genetic mechanisms associated with carcass merit traits in beef cattle
Source: Sci Rep. 2022 Mar 1;12:3389. doi: 10.1038/s41598-022-06567-z (PMC8888742; doi:10.1038/s41598-022-06567-z)
Supplement: Supplementary file 4 — Supplementary Information 4. [file 41598_2022_6567_MOESM4_ESM.docx]

**Integrative analyses of genomic and metabolomic data reveal genetic mechanisms associated with carcass merit traits in beef cattle**

Jiyuan Li^1^, Yining Wang^1,2^, Robert Mukiibi^3^, Brian Karisa^4^, Graham S. Plastow^1^*, Changxi Li^1,2^*

^1^Department of Agriculture, Food & Nutritional Science, University of Alberta, Edmonton, Alberta, Canada, T6G 2P5, ^2^Lacombe Research and Development Centre, Agriculture and Agri-food Canada, Lacombe, Alberta, Canada, T4L 1W1, ^3^The Roslin Institute and Royal (Dick) School of Veterinary Studies, University of Edinburgh, Edinburgh, Scotland, UK, EH25 9RG, ^4^Ministry of Agriculture and Forestry, Edmonton, Alberta, Canada, T6N 1E4

Correspondence and requests for materials should be addressed to Changxi Li (changxi.li@agr.gc.ca ) or Graham Plastow (plastow@ualberta.ca)

**
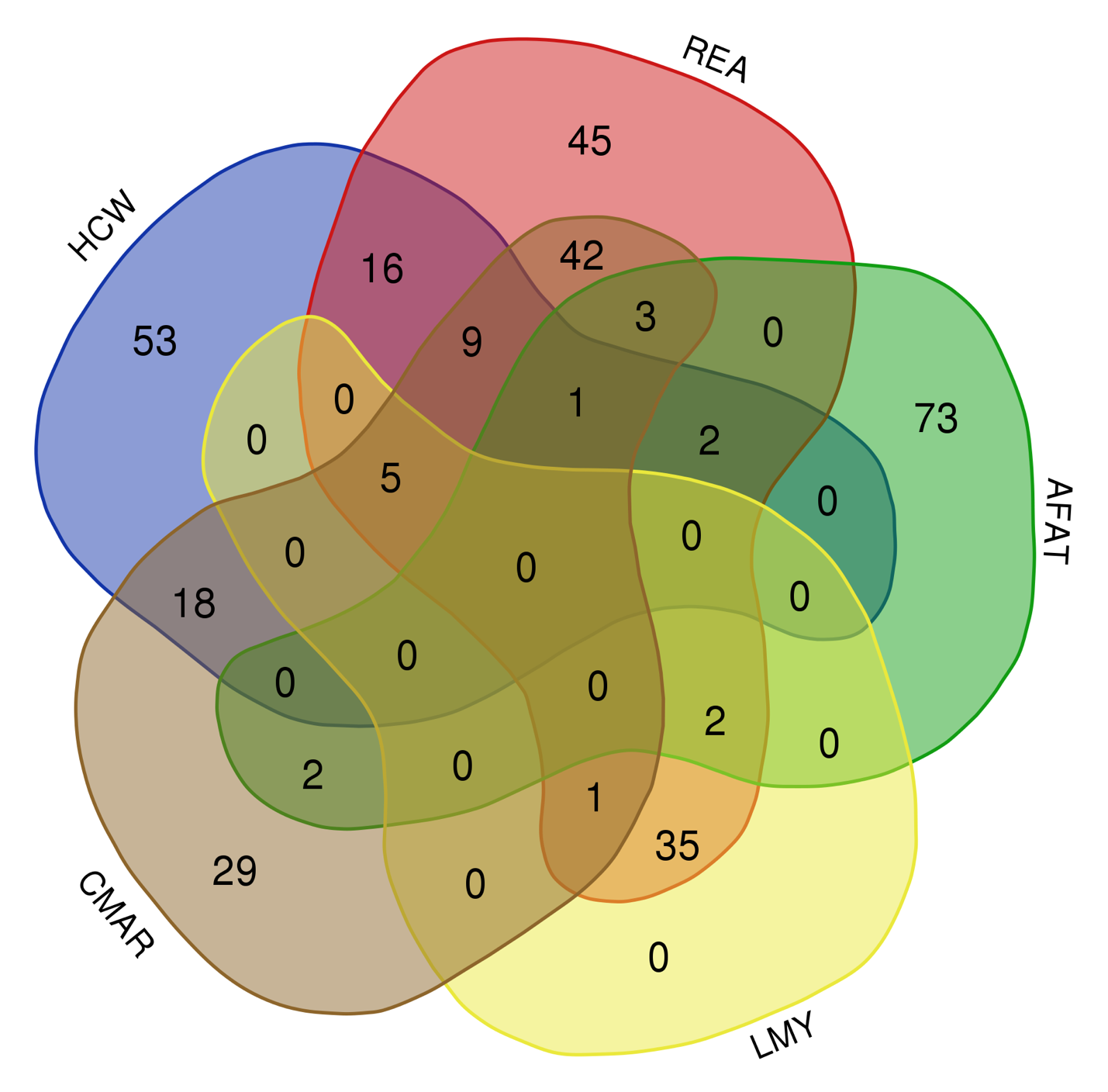
**

**Figure S1** Uniquely common candidate genes for carcass merit traits in a beef cattle multibreed population


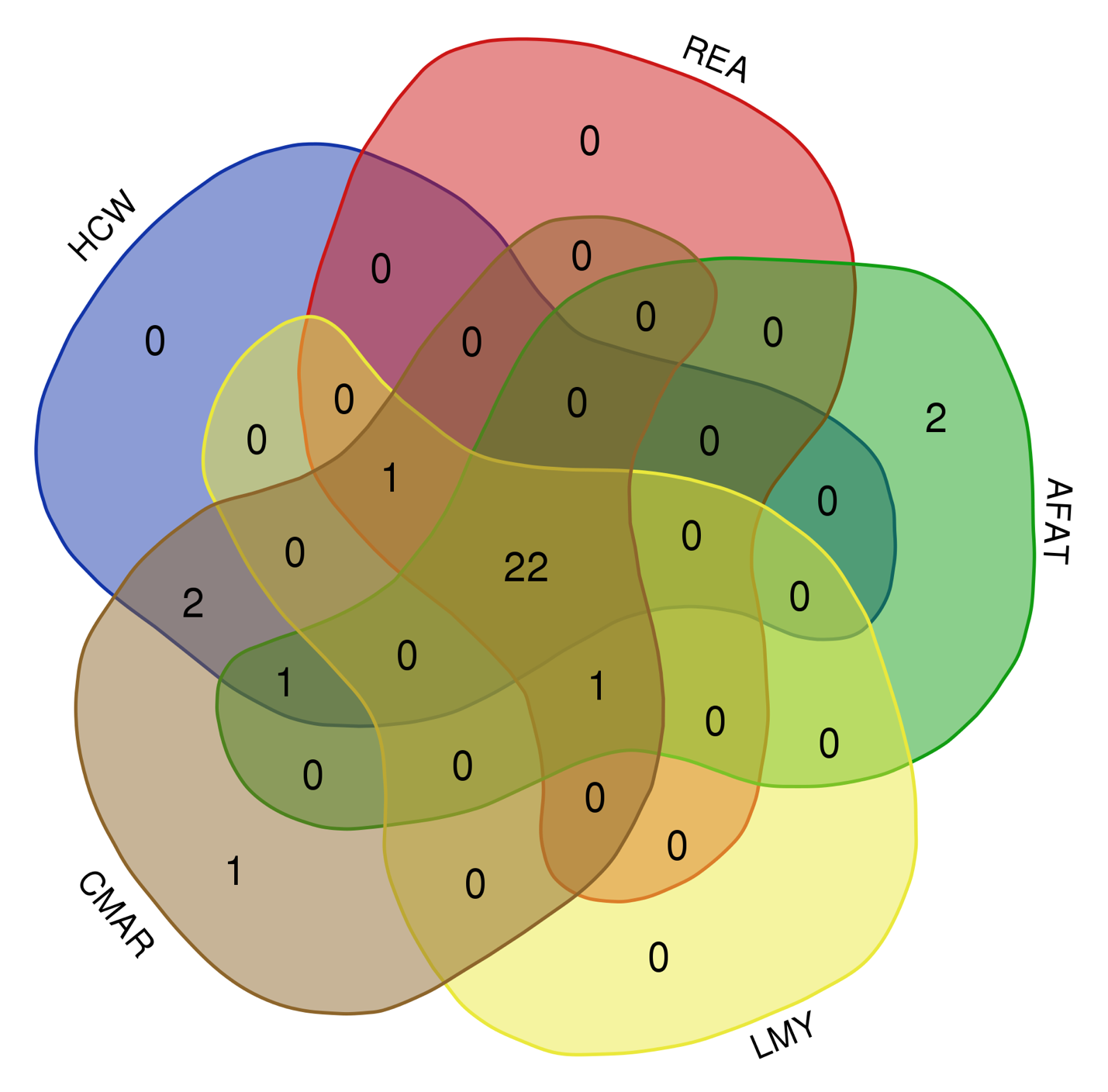


**Figure S2** Uniquely common biological functions for carcass merit traits in a beef cattle multibreed population


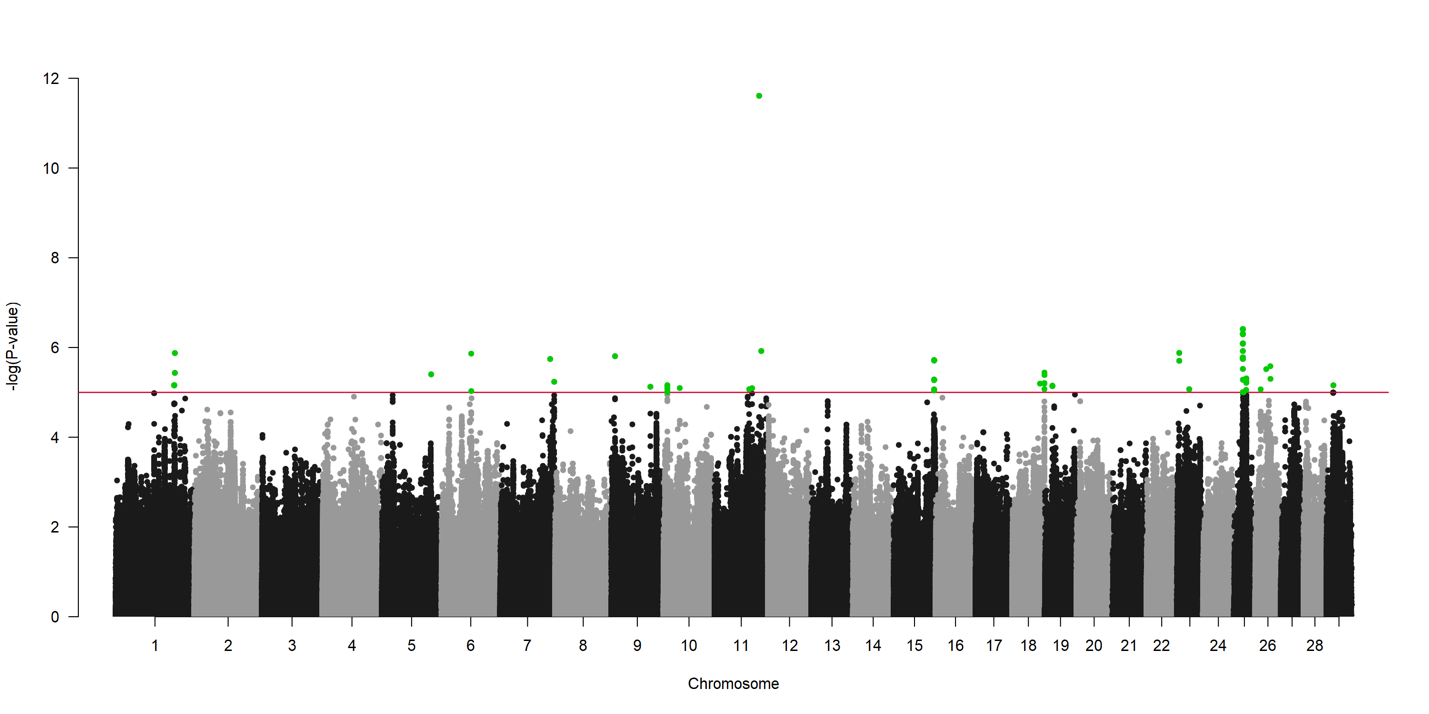


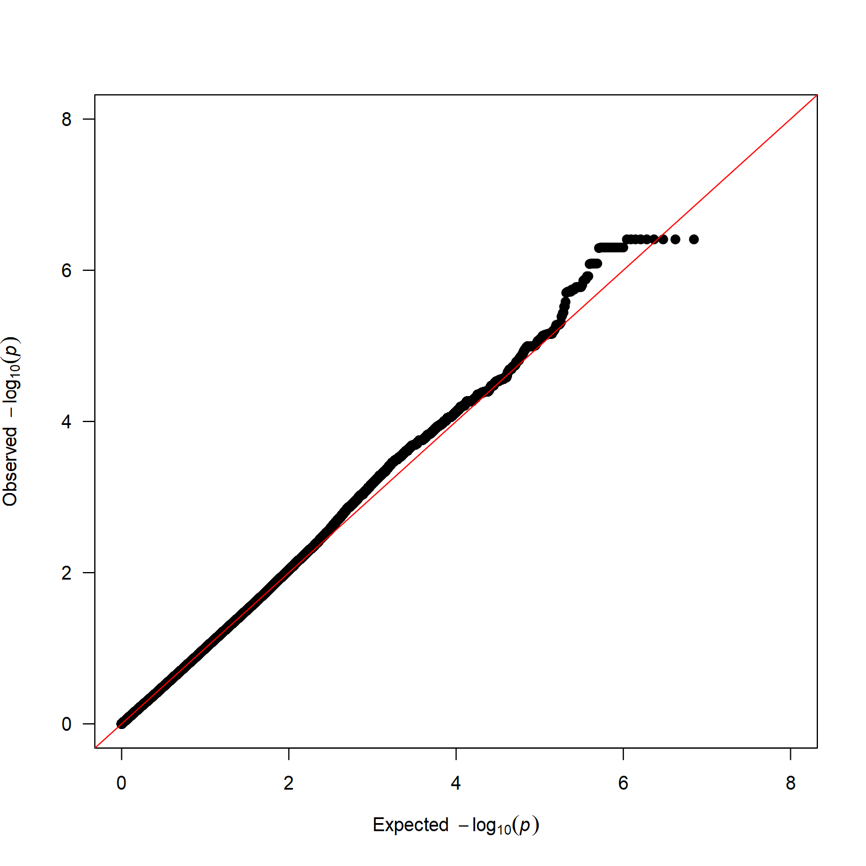


**Figure S3** Manhattan plot and QQ plot for acetic acid


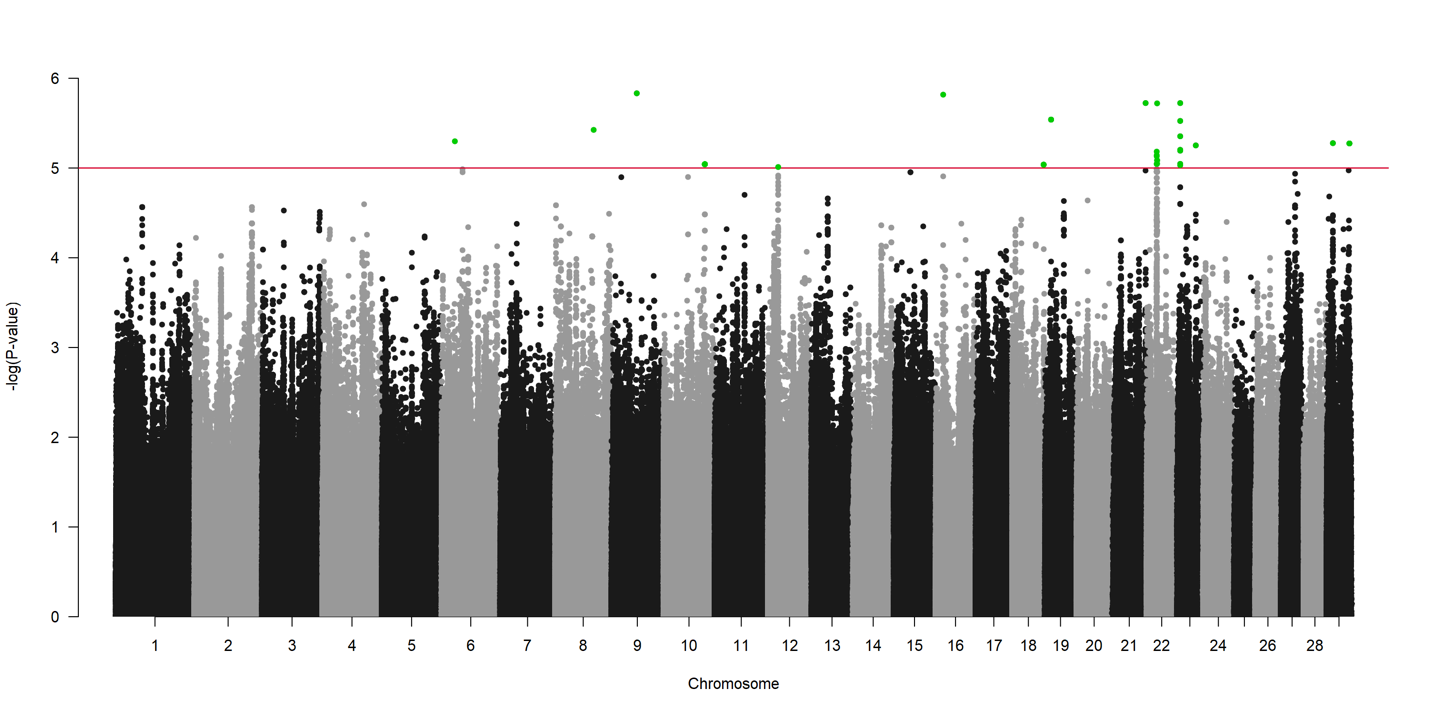


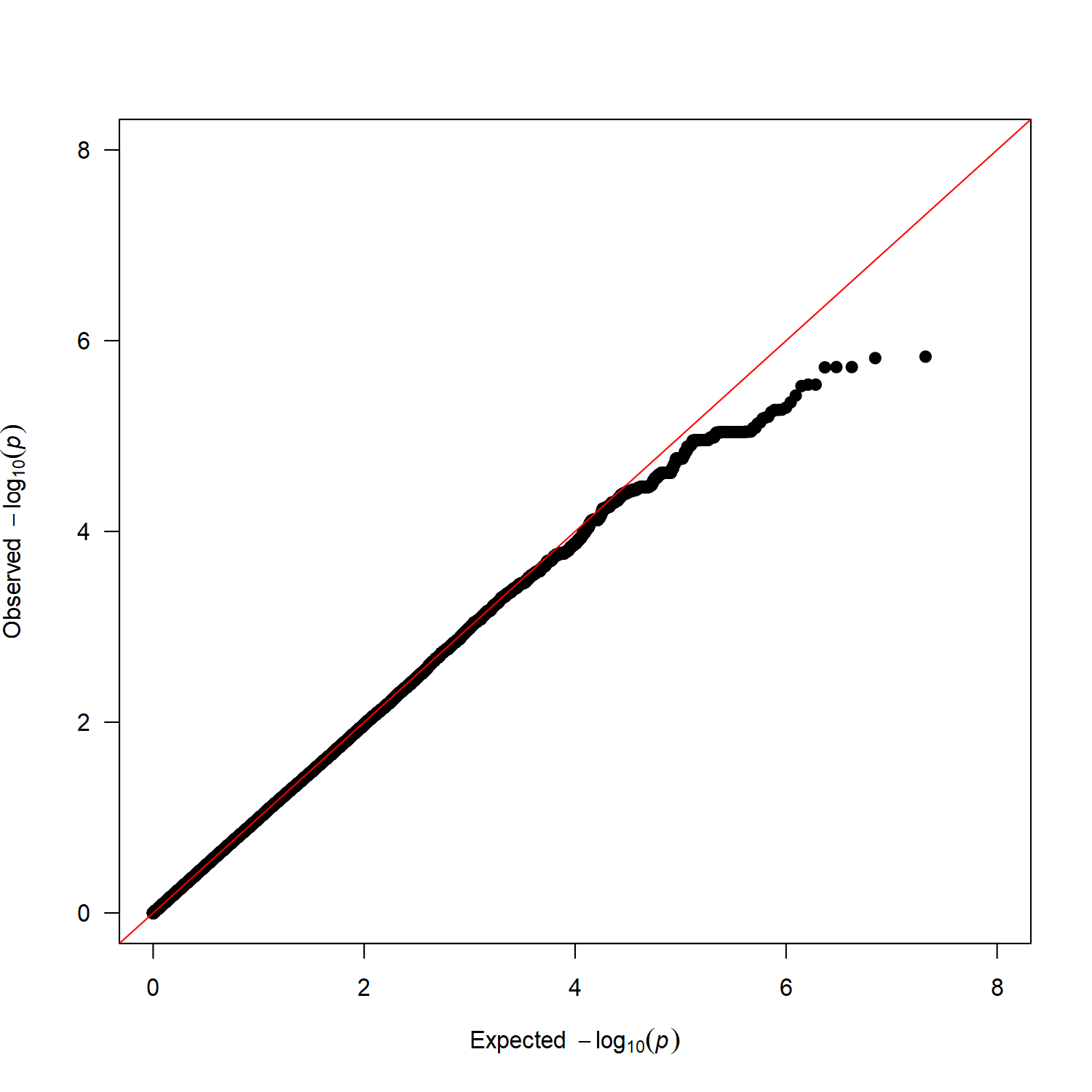


**Figure S4** Manhattan plot and QQ plot for citric acid


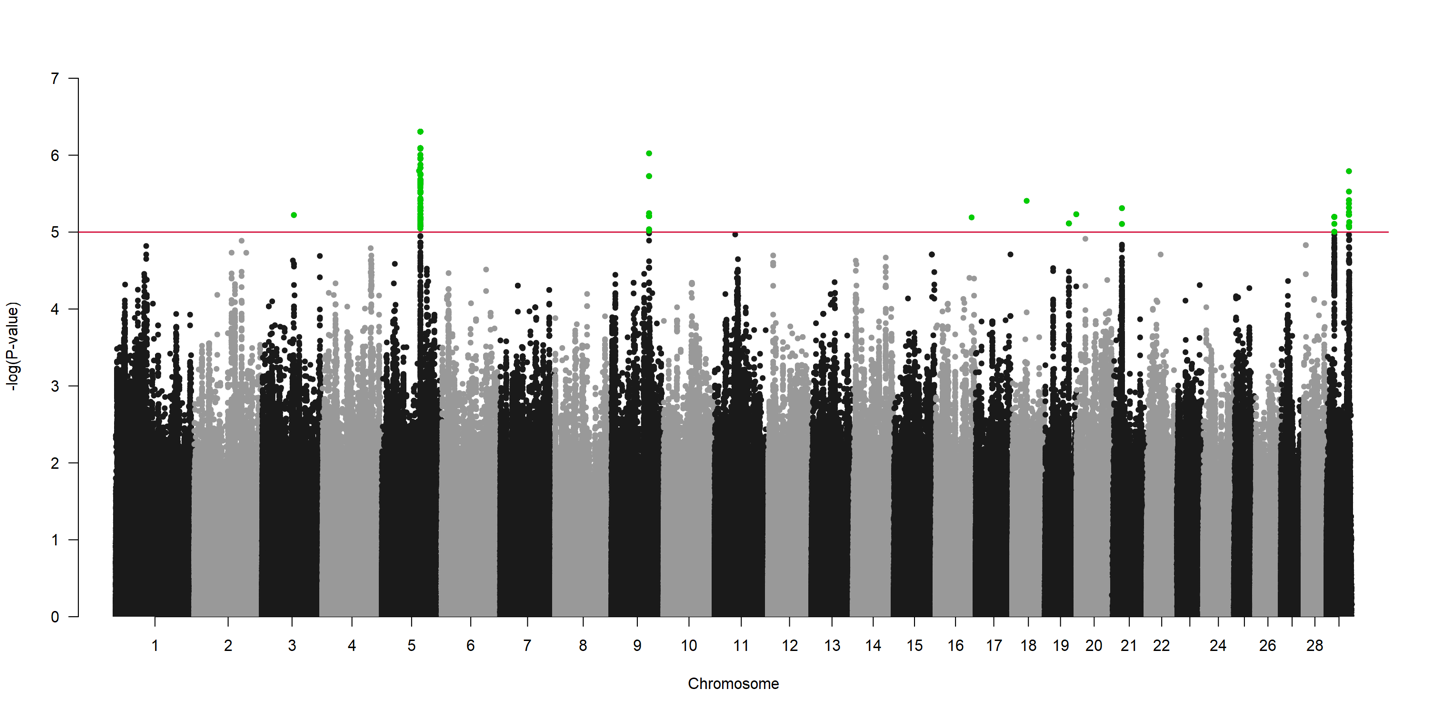


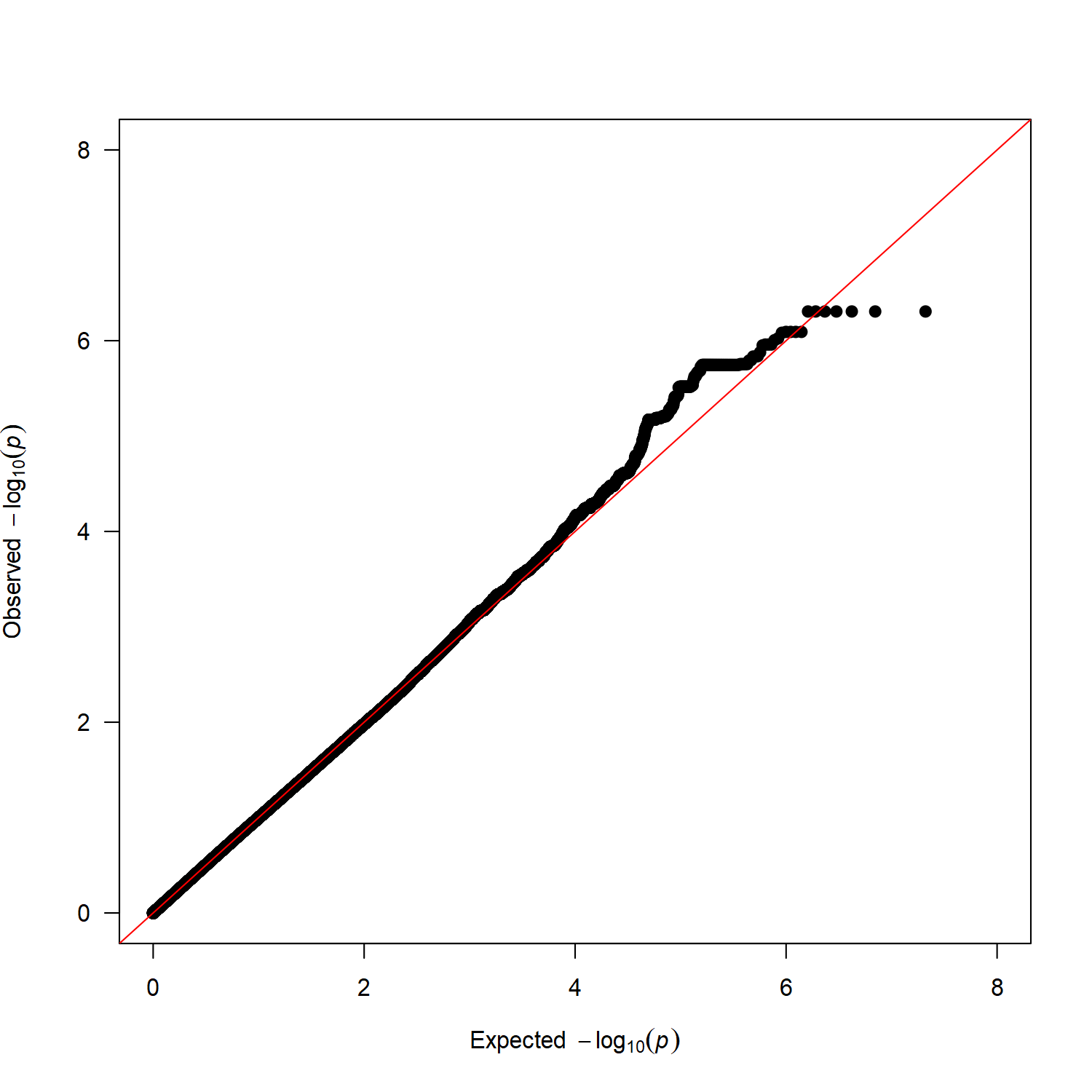


**Figure S5** Manhattan plot and QQ plot for choline


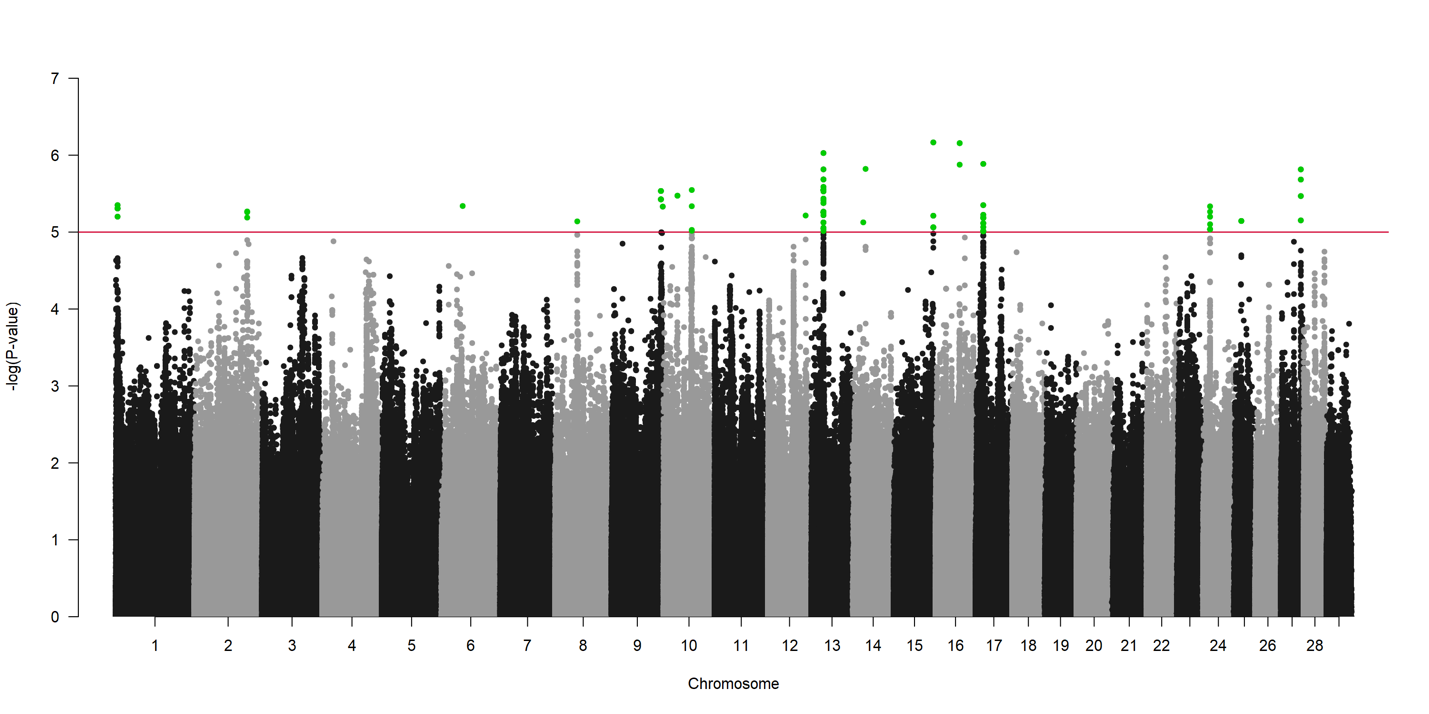


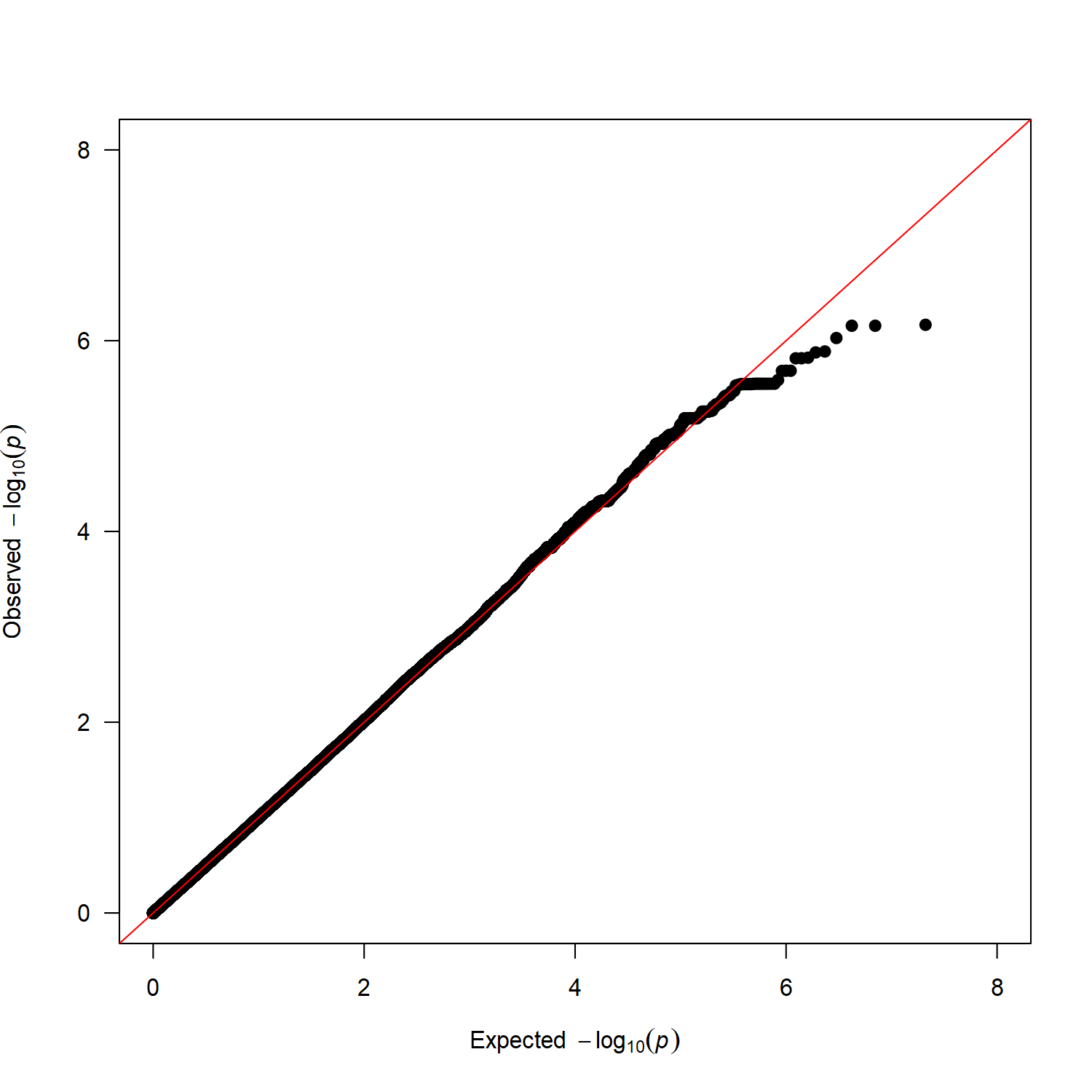


**Figure S6** Manhattan plot and QQ plot for D-glucose


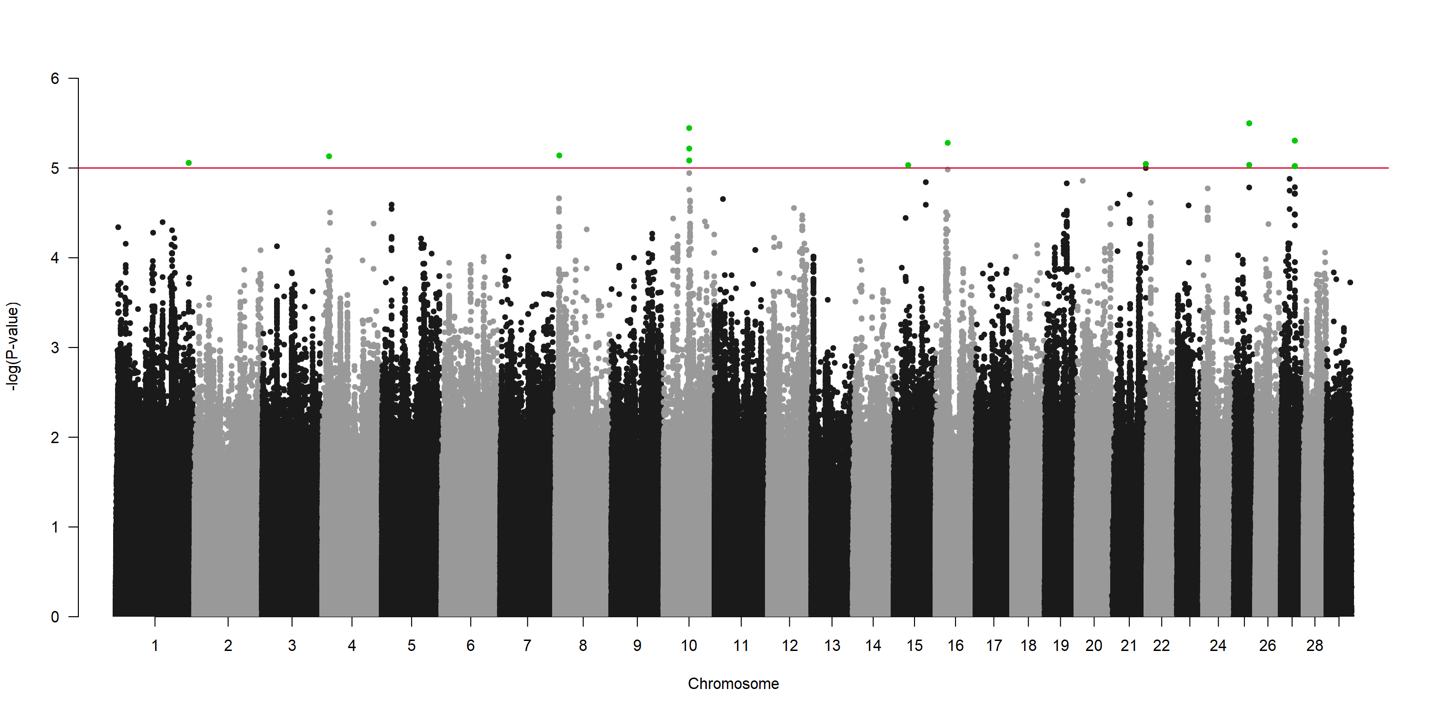


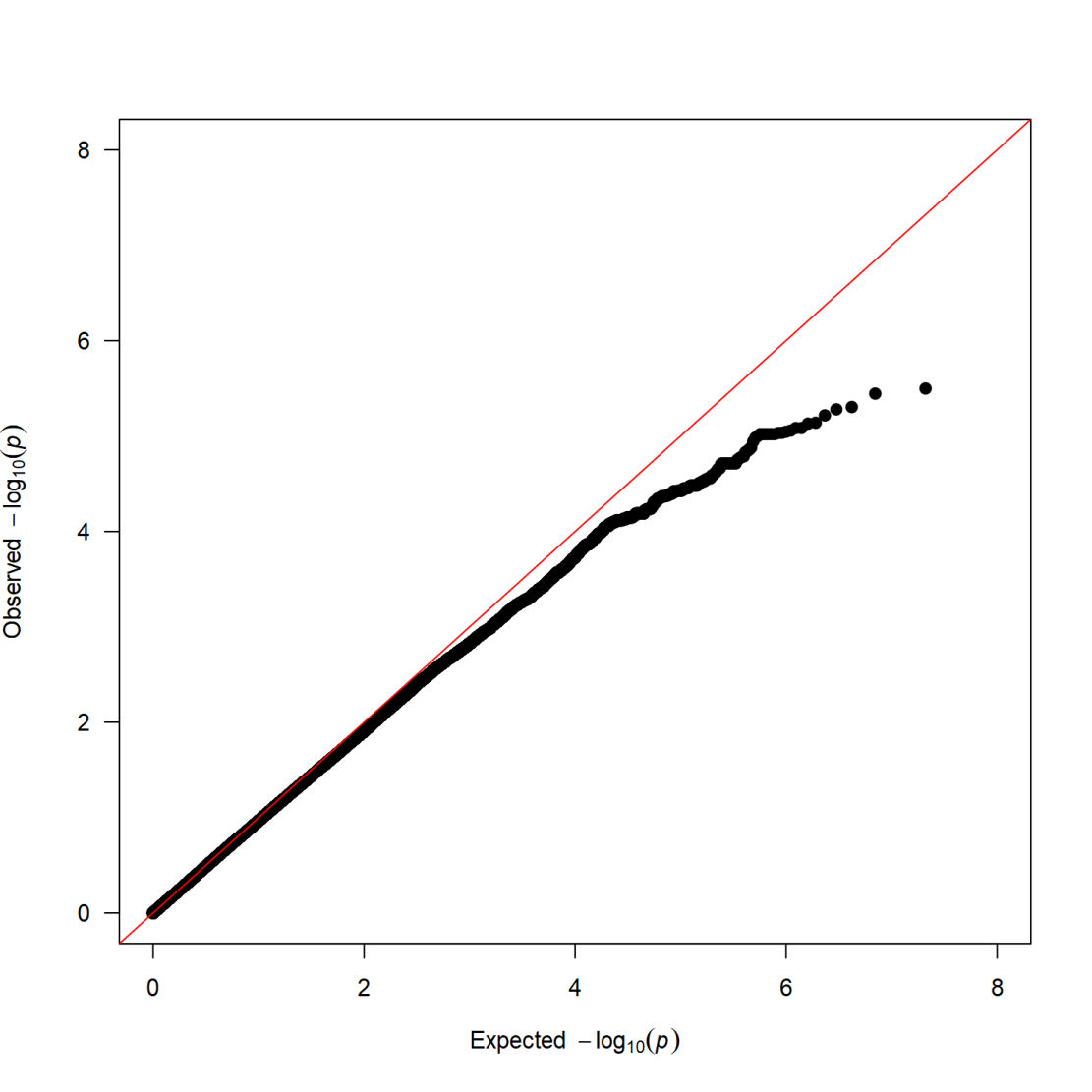


**Figure S7** Manhattan plot and QQ plot for glycine


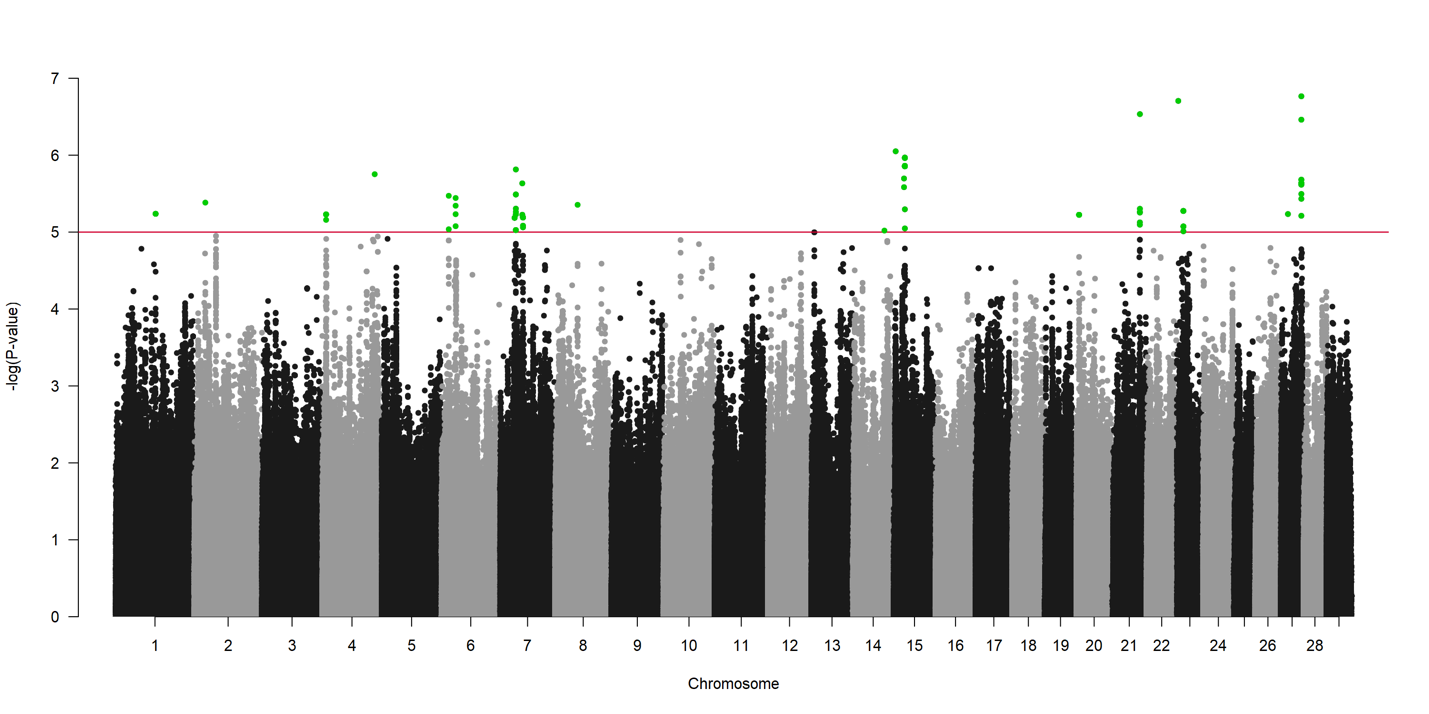


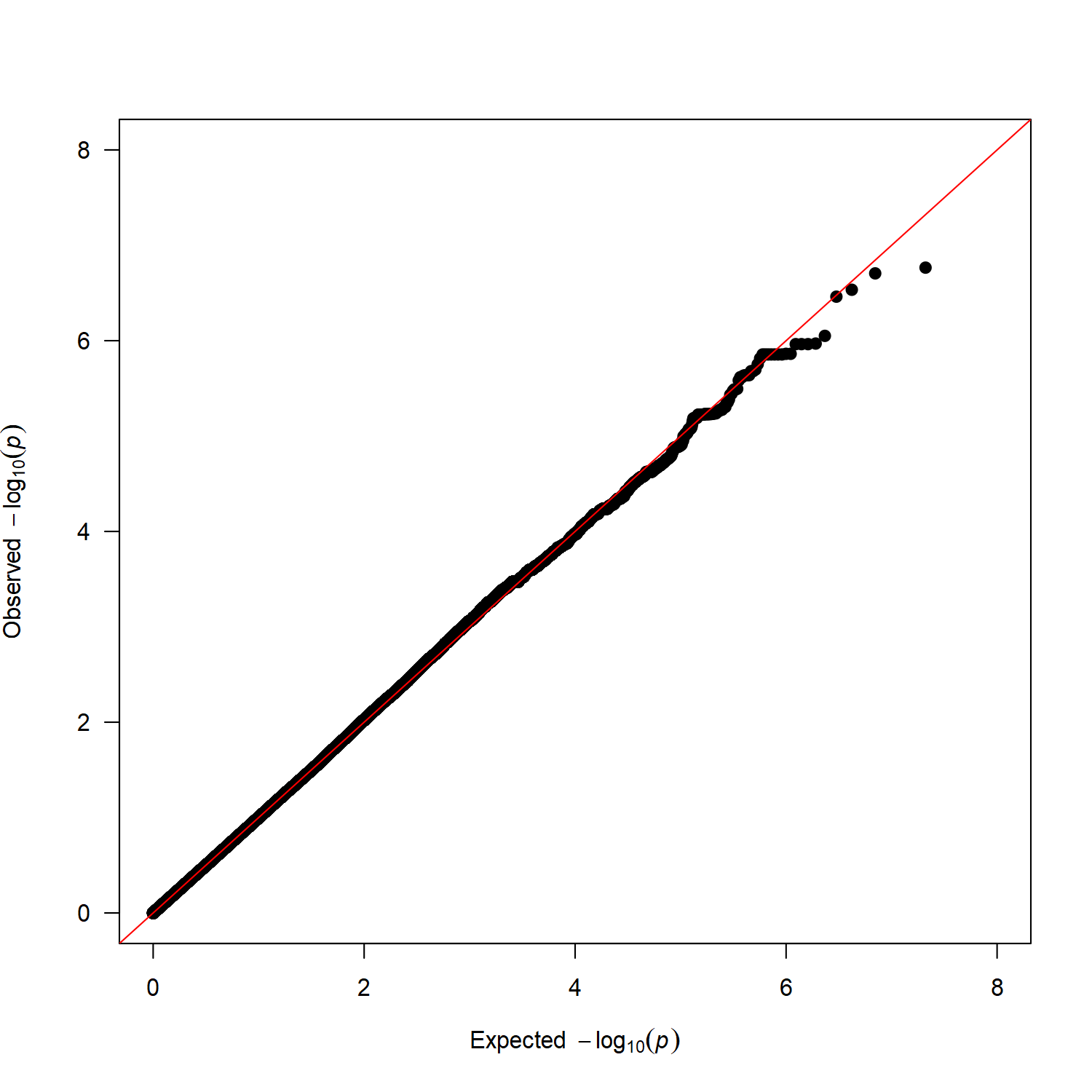


**Figure S8** Manhattan plot and QQ plot for glycerol


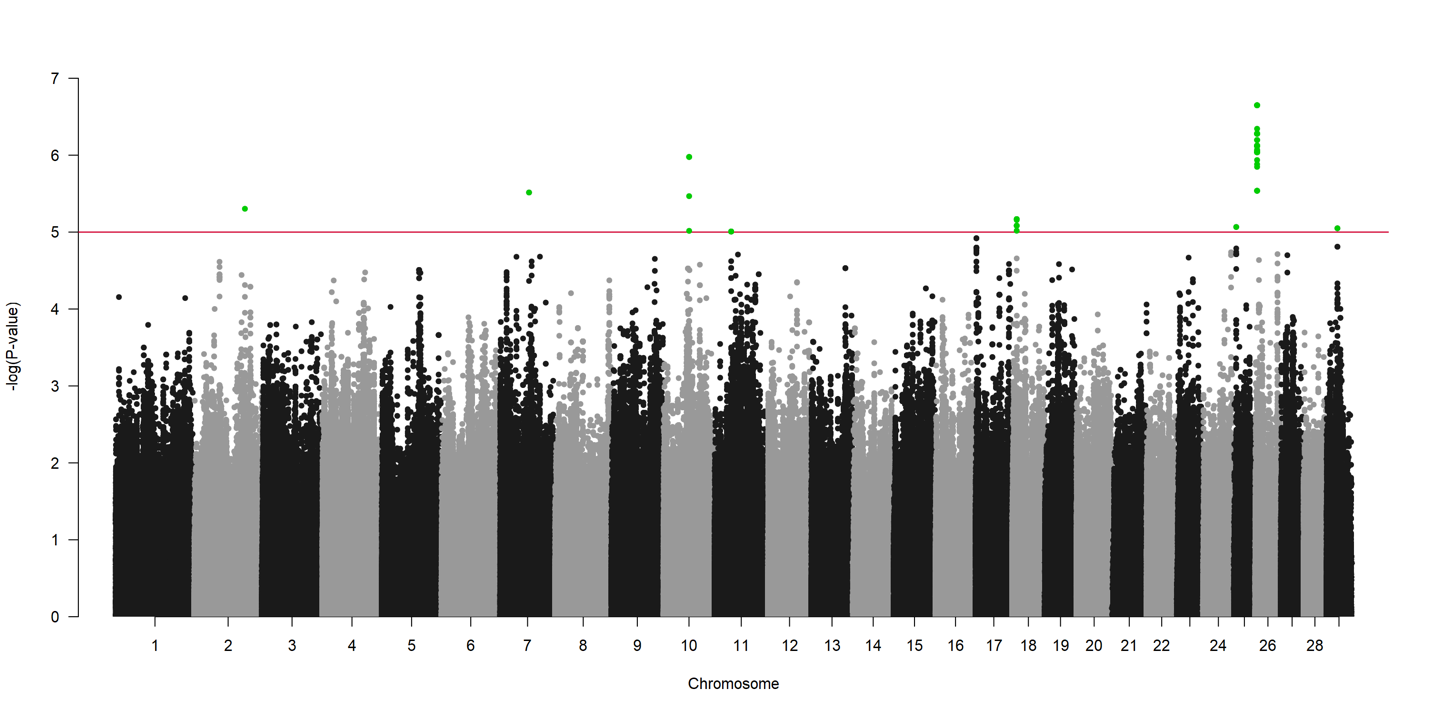


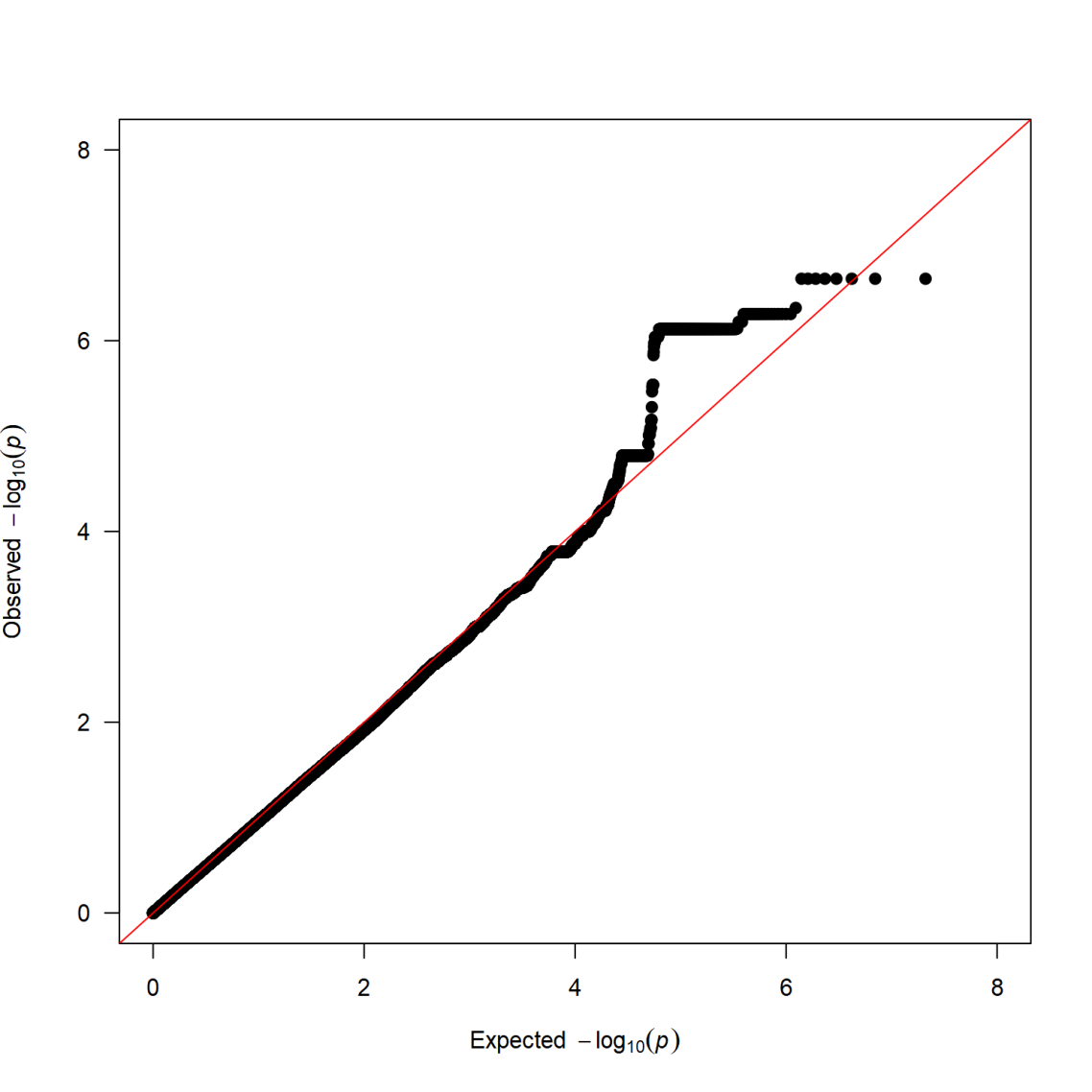


**Figure S9** Manhattan plot and QQ plot for fumaric acid


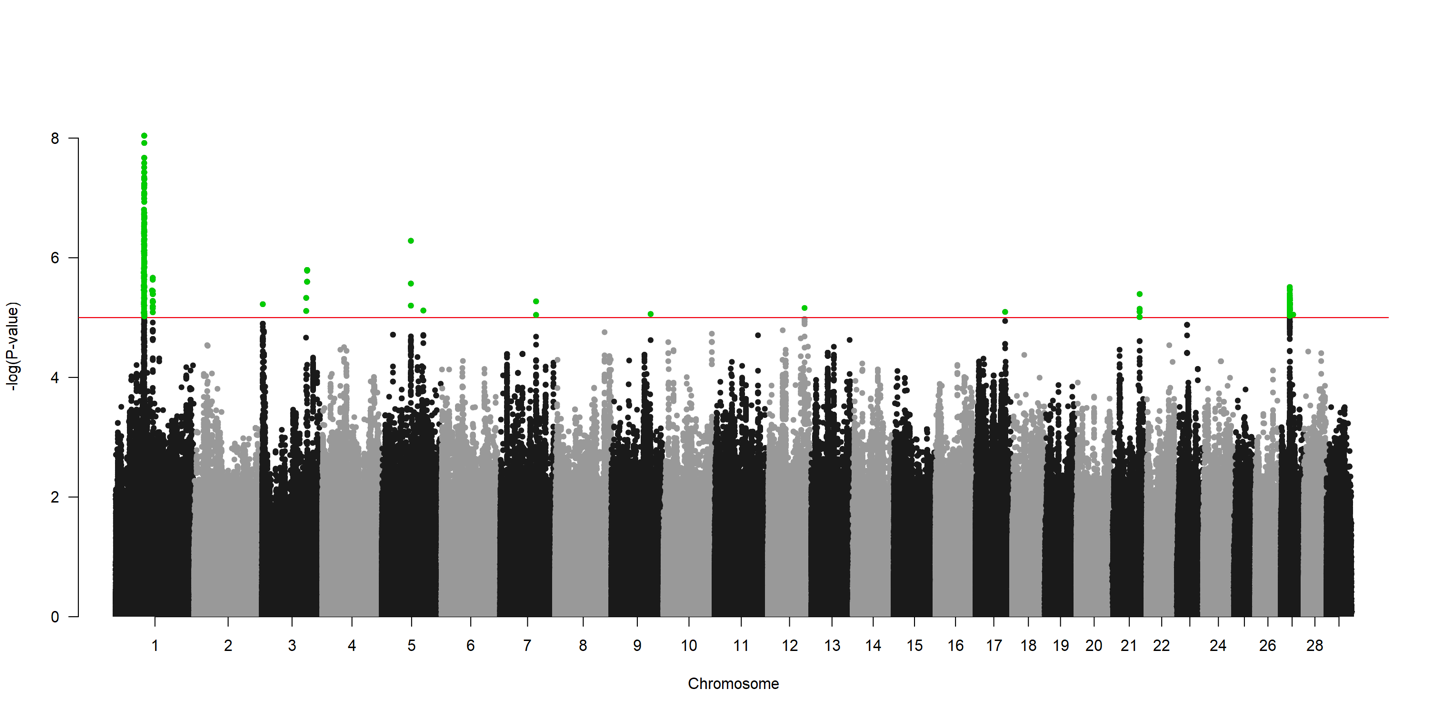


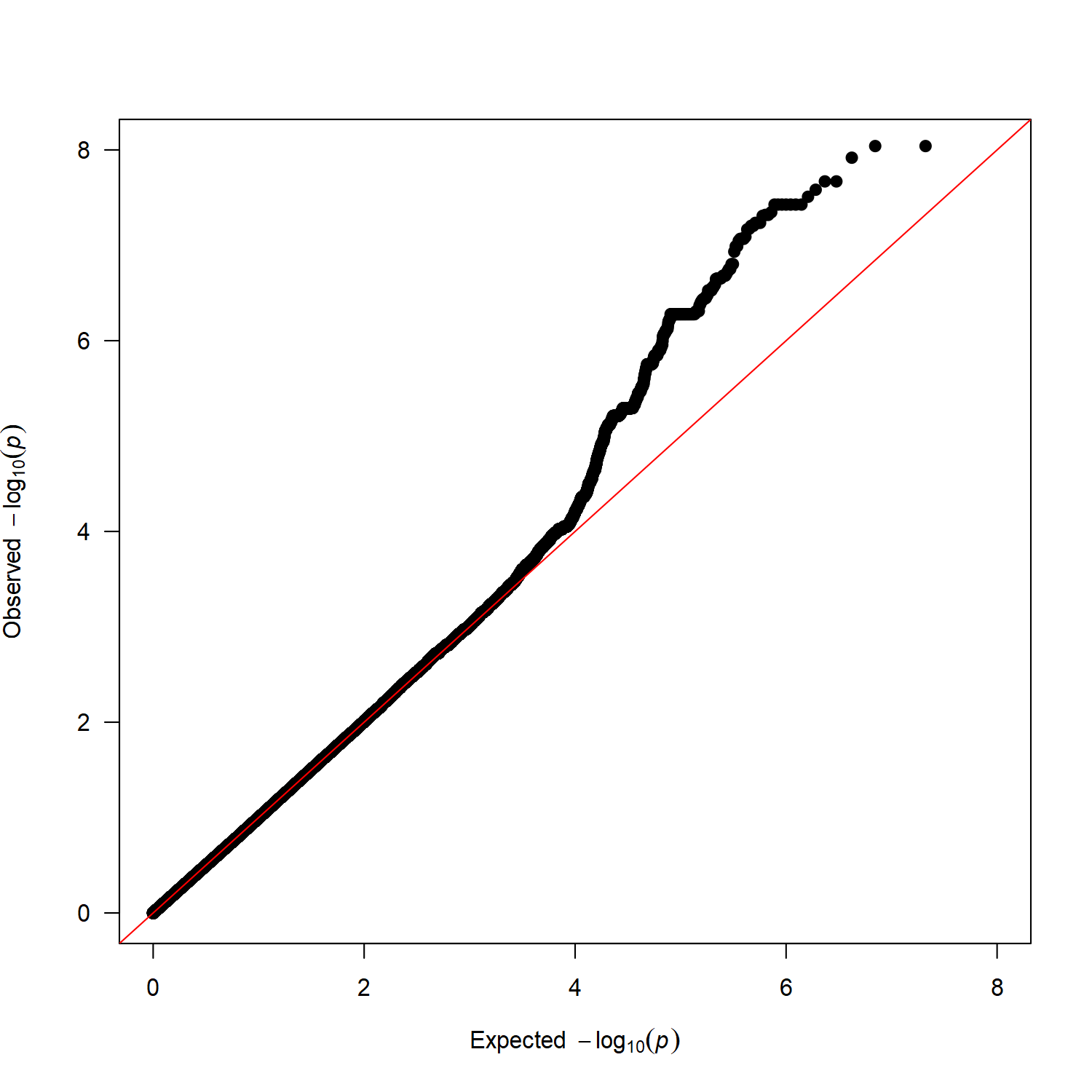


**Figure S10** Manhattan plot and QQ plot for lysine


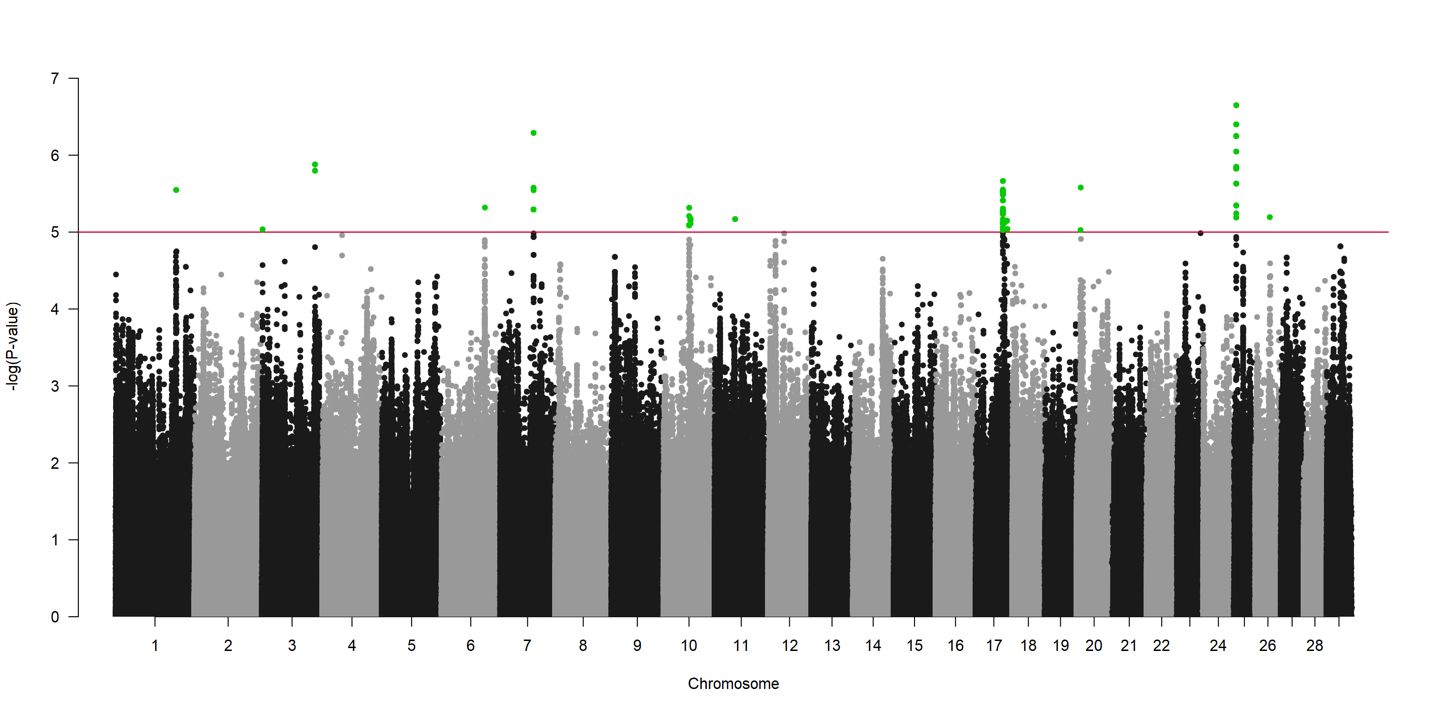


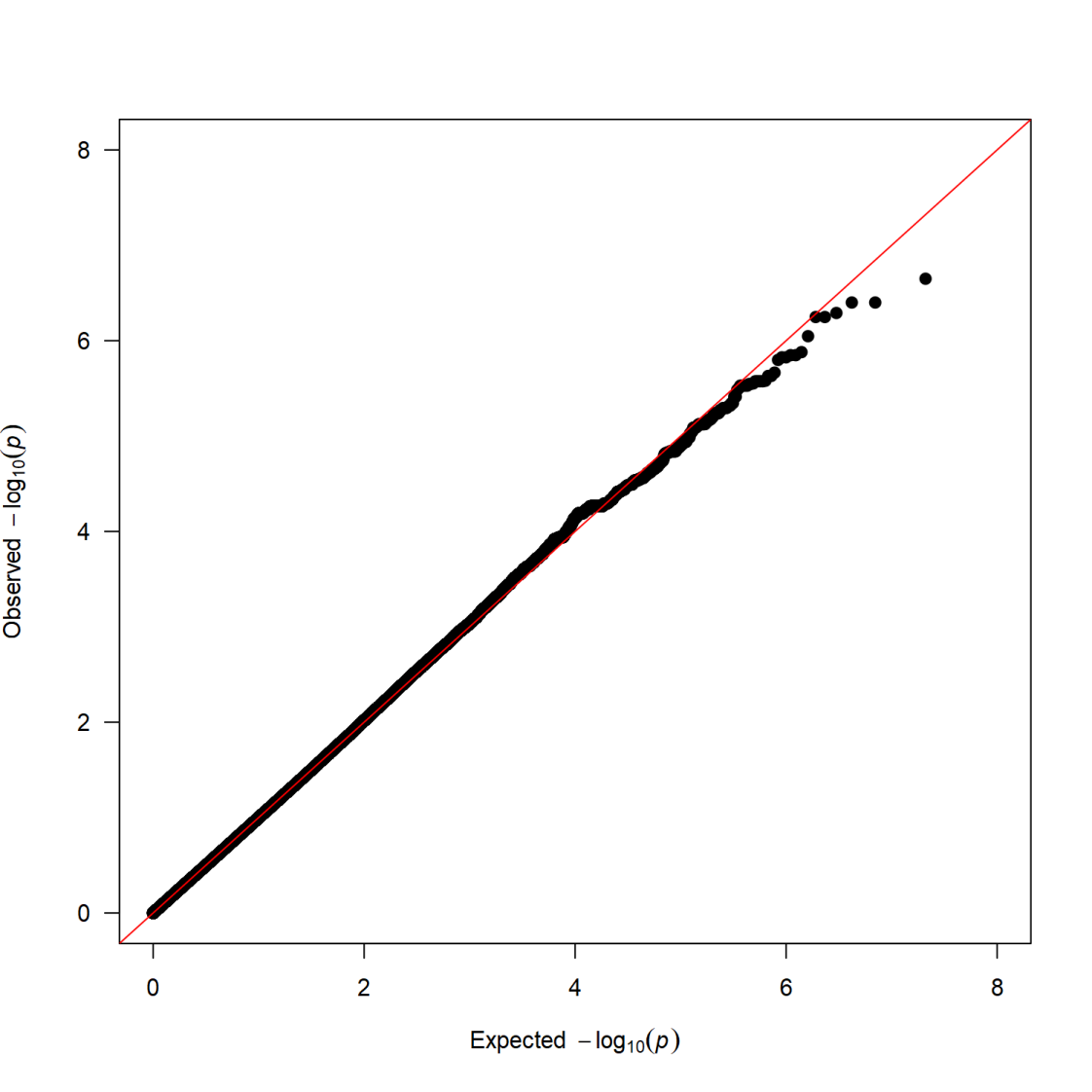


**Figure S11** Manhattan plot and QQ plot for L-lactic acid


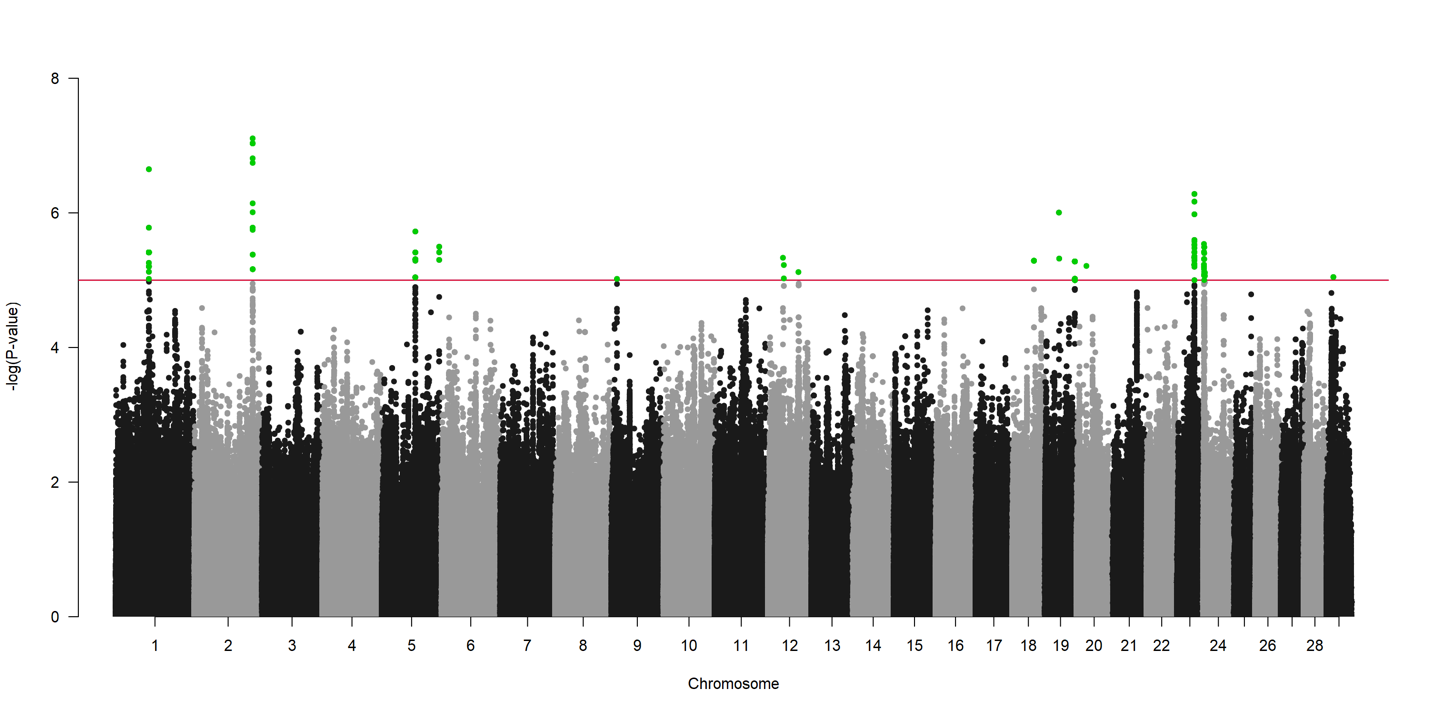


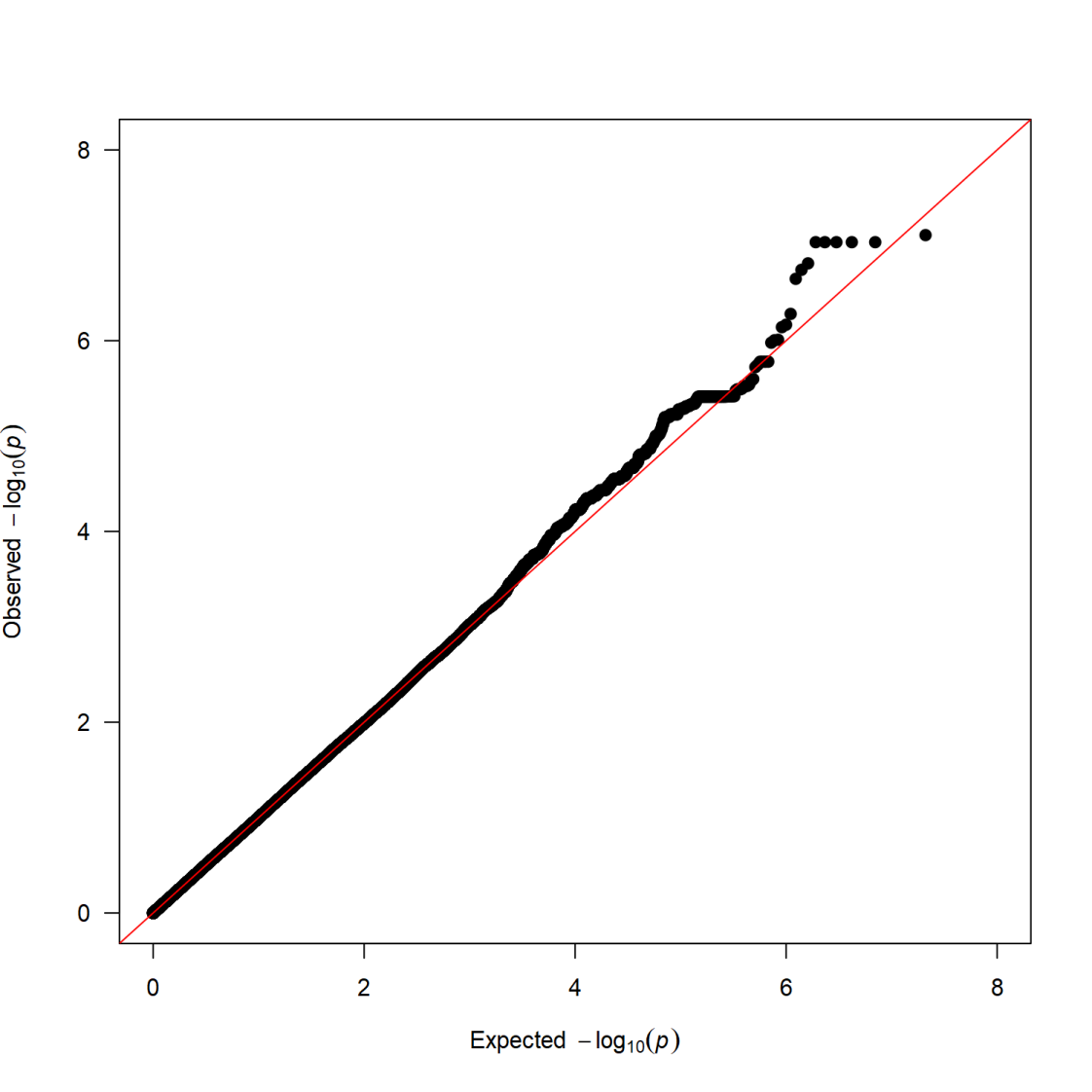


**Figure S12** Manhattan plot and QQ plot for pyruvic acid


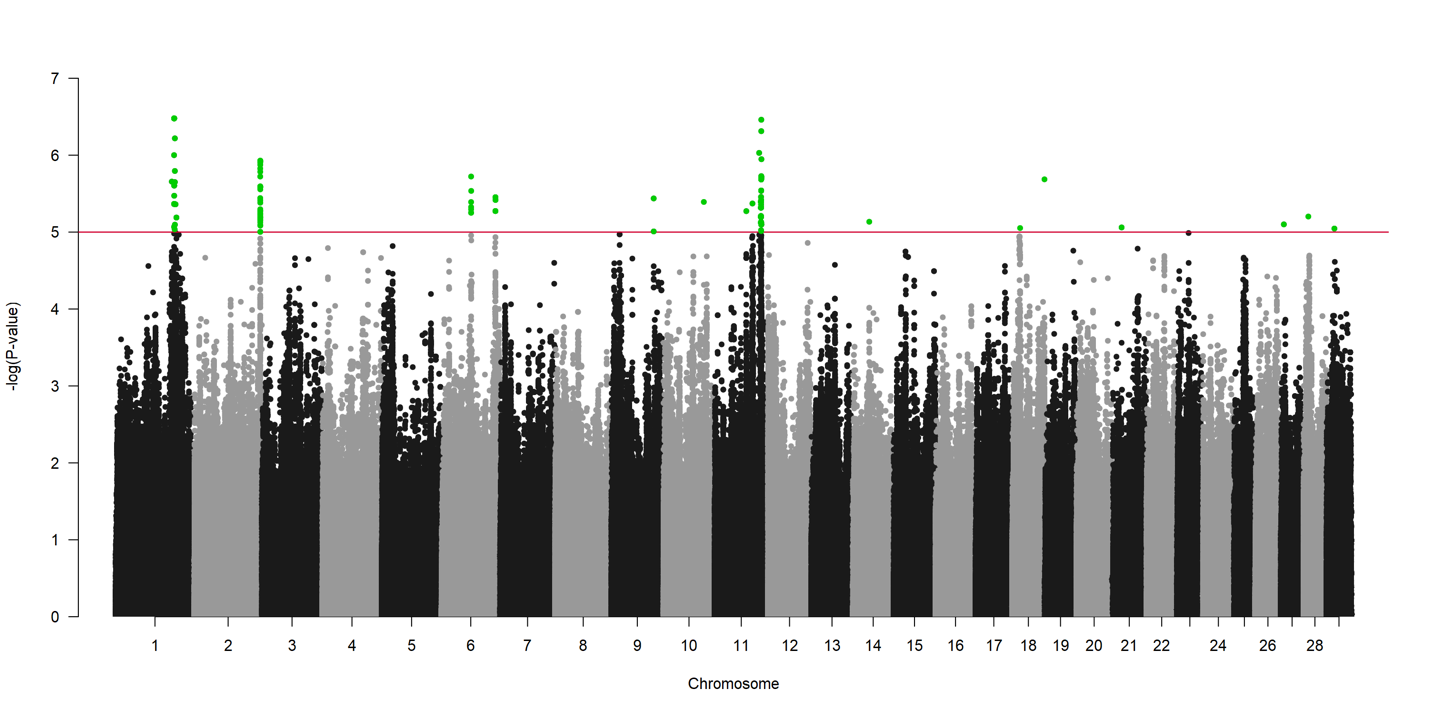


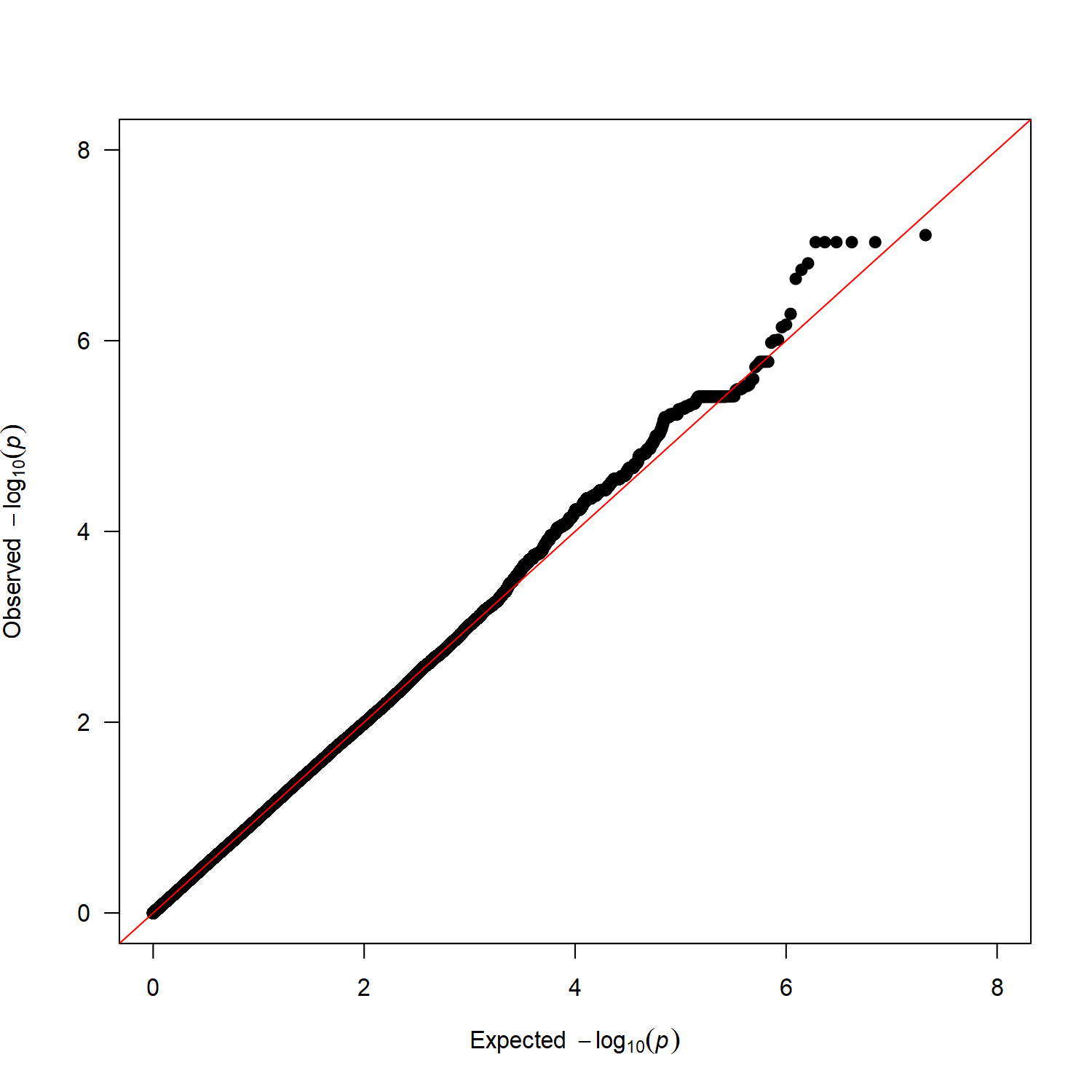


**Figure S13** Manhattan plot and QQ plot for succinic acid


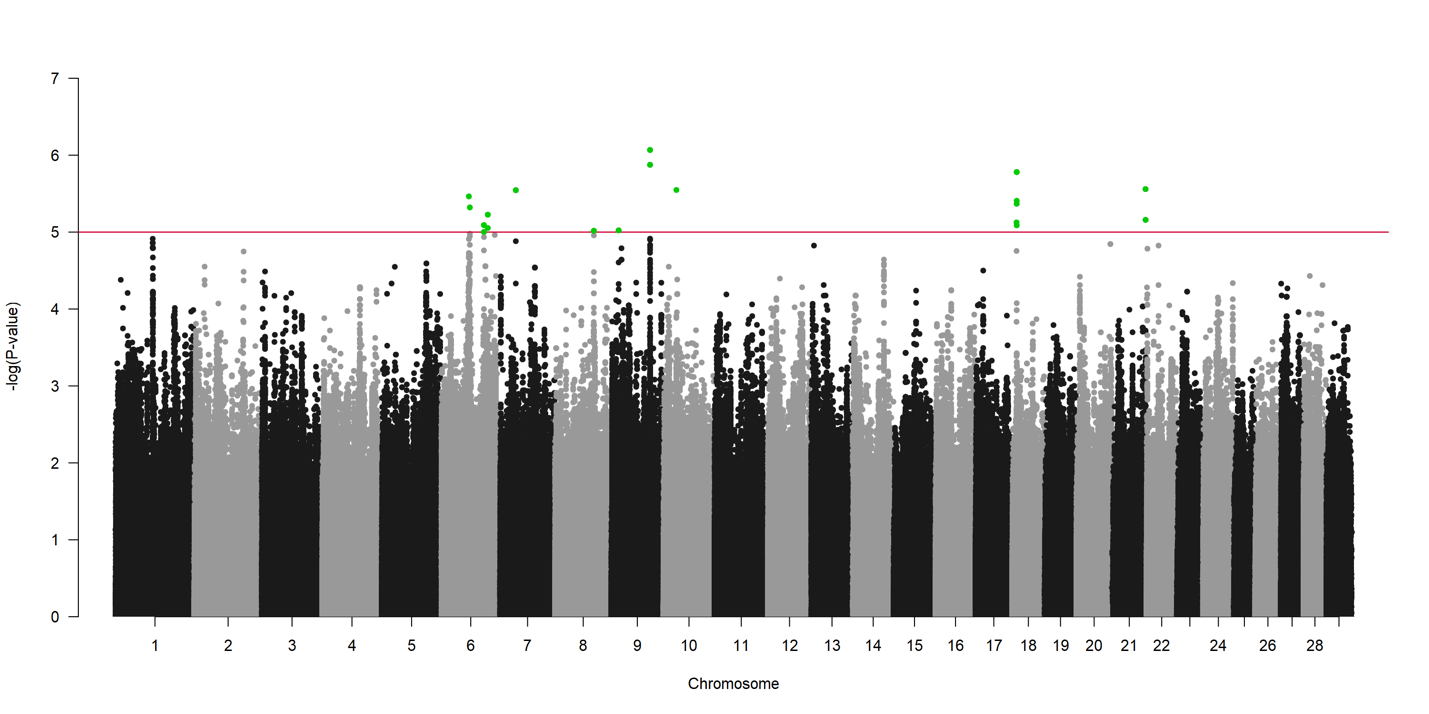


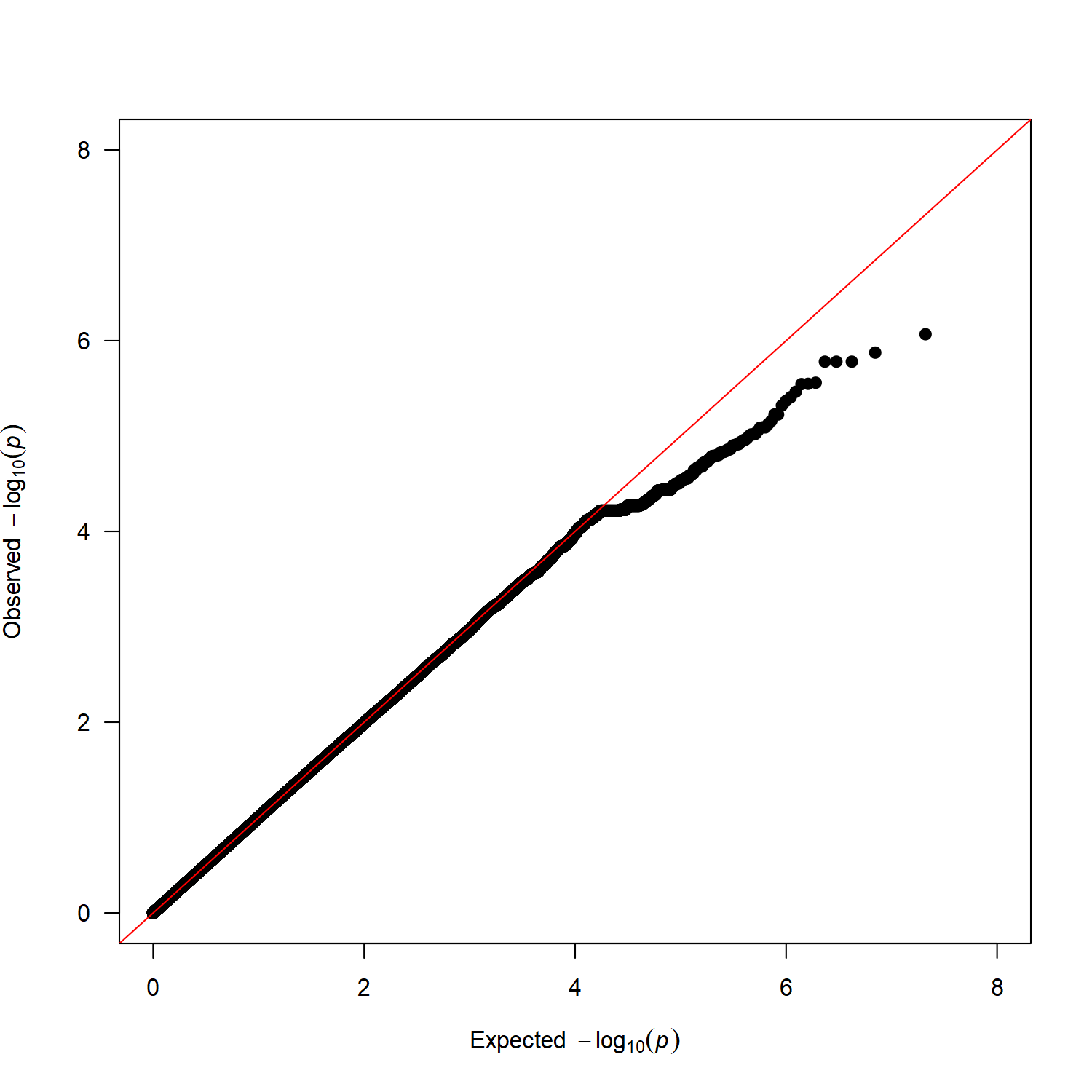


**Figure S14** Manhattan plot and QQ plot for 3-hydroxybutyric acid


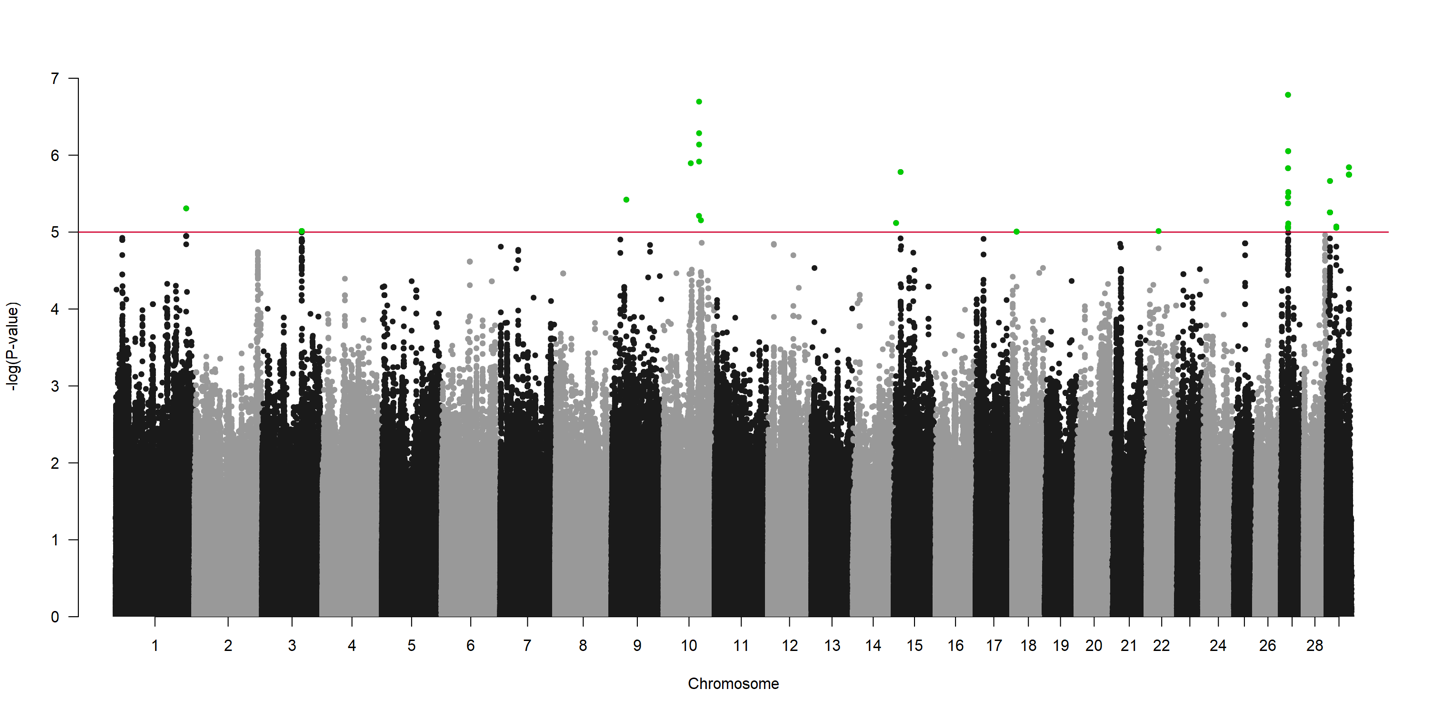


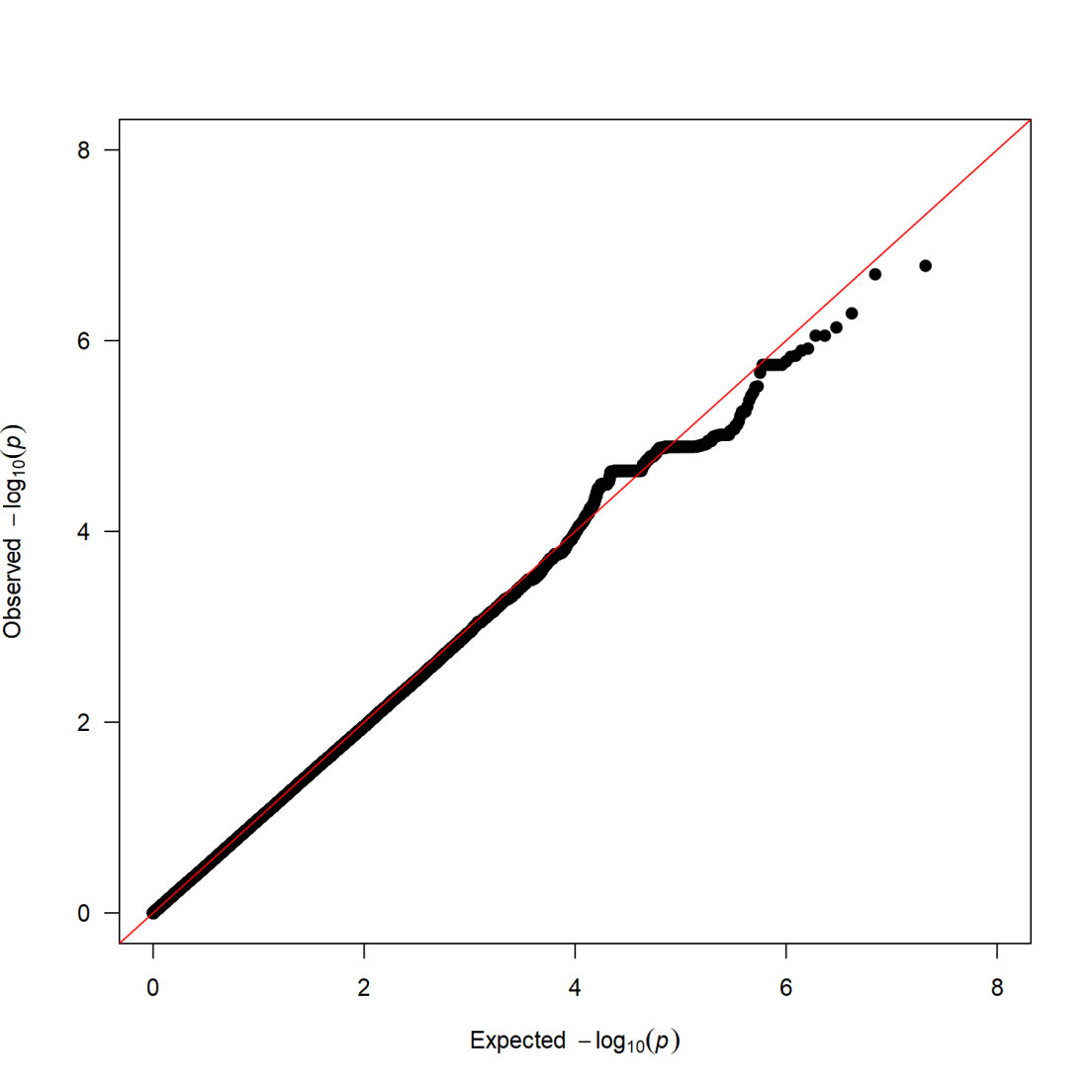


**Figure S15** Manhattan plot and QQ plot for creatinine


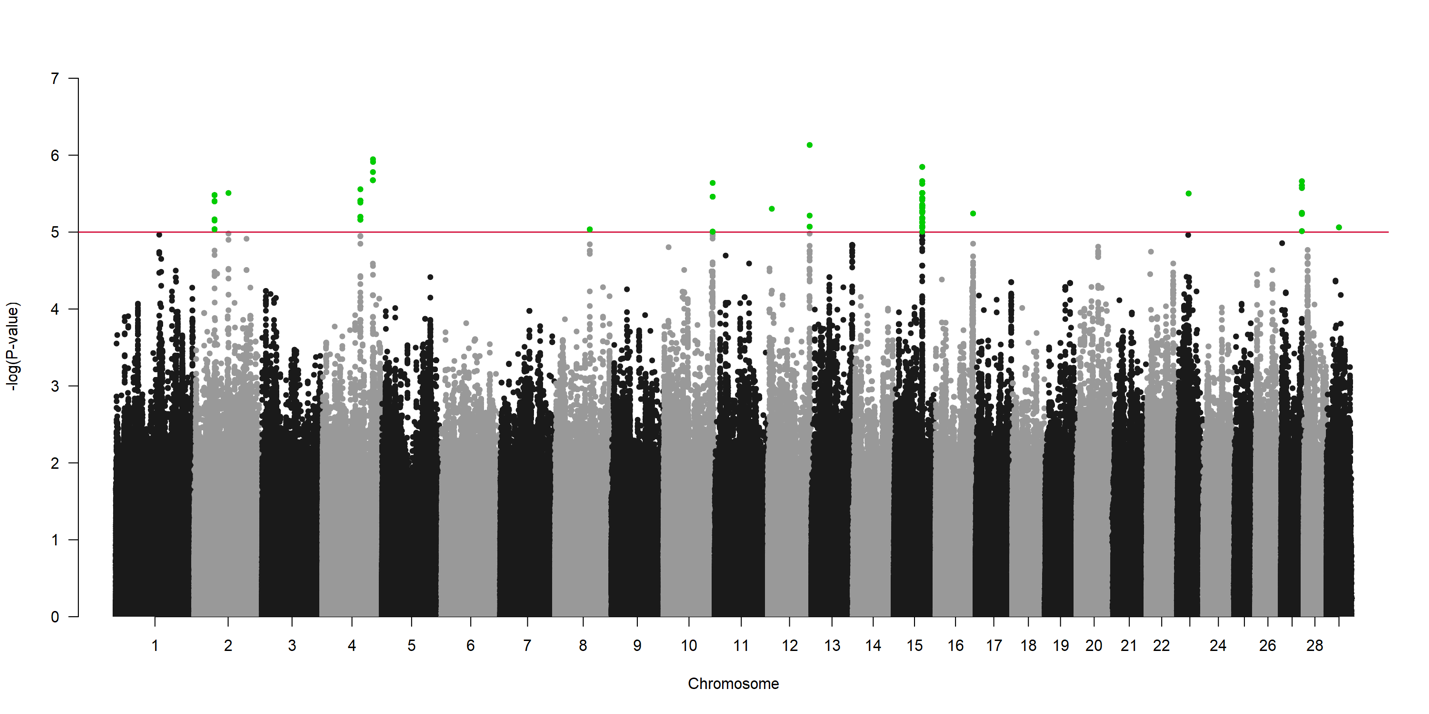


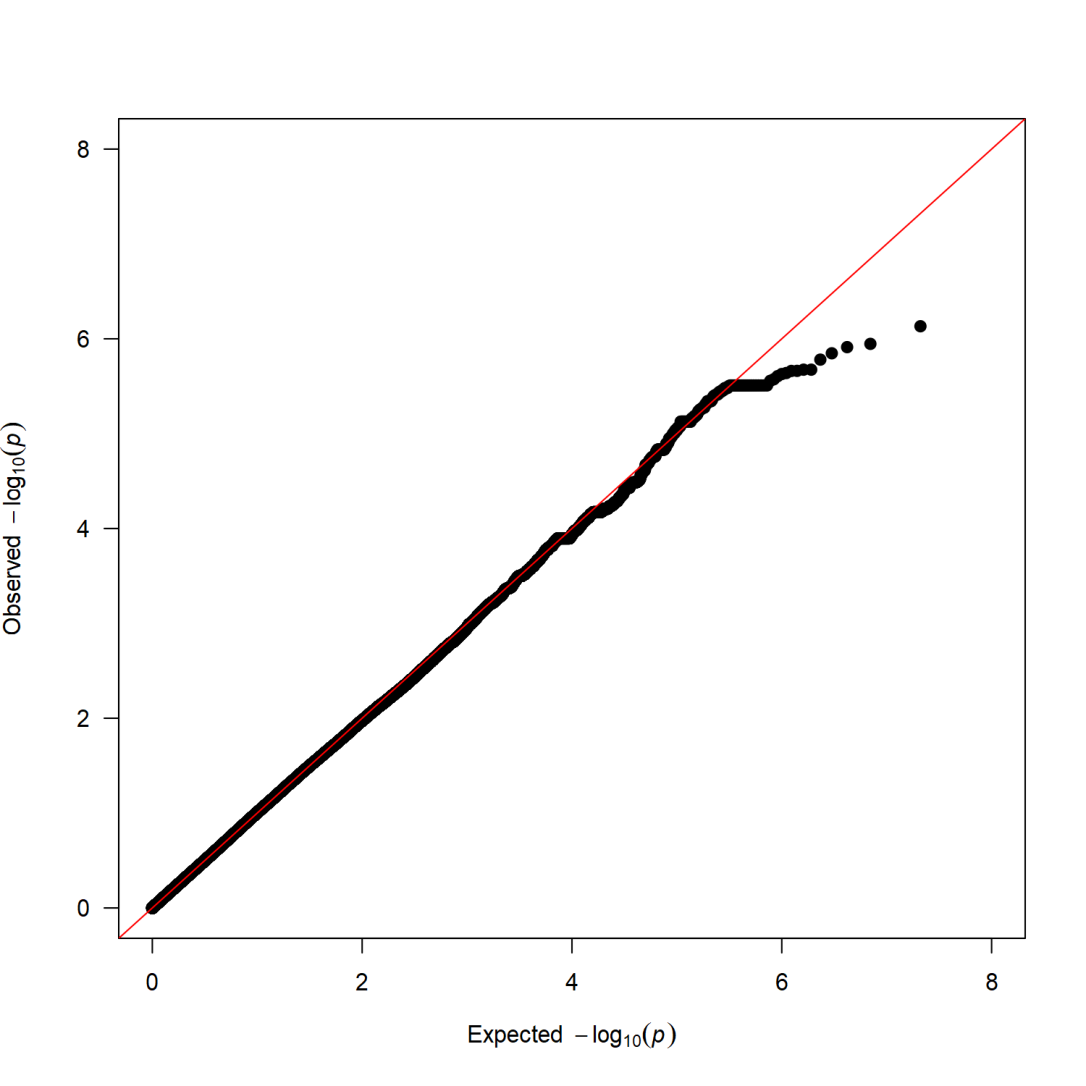


**Figure S16** Manhattan plot and QQ plot for L-glutamine


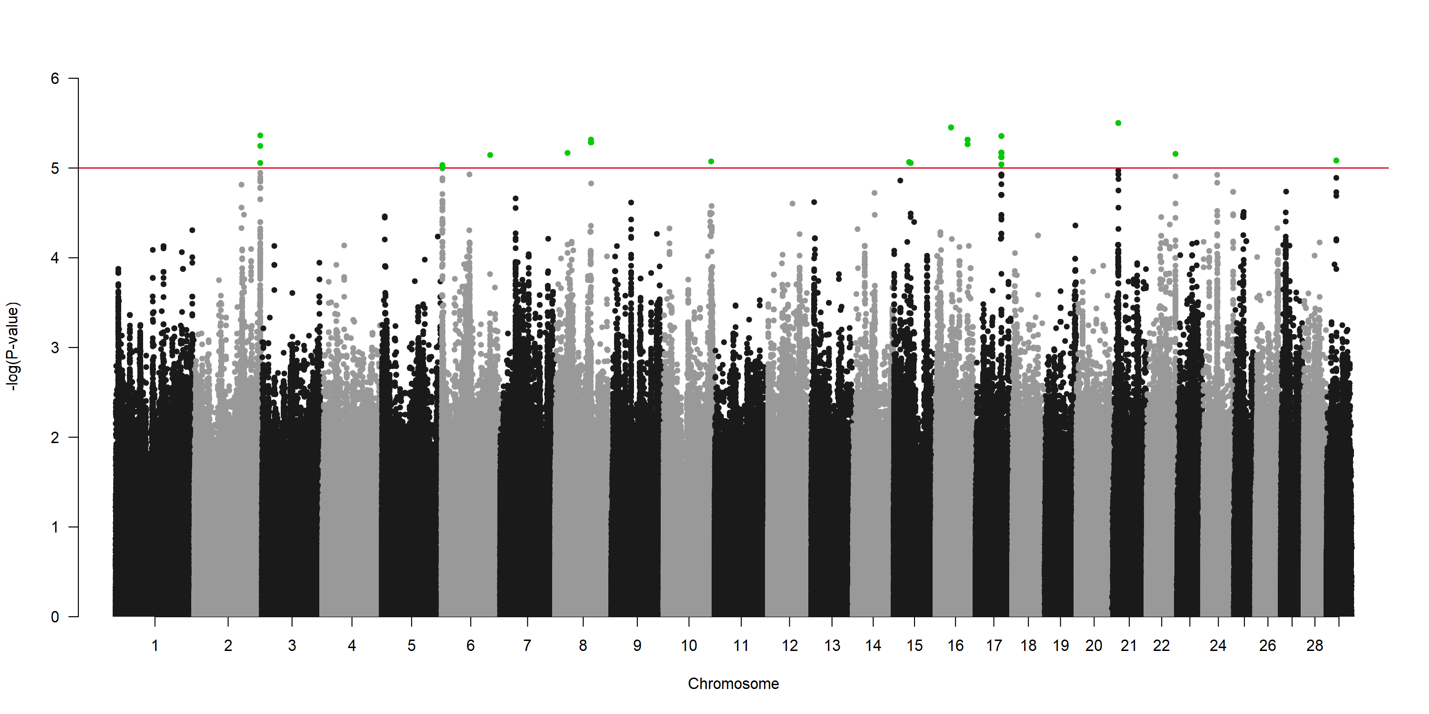


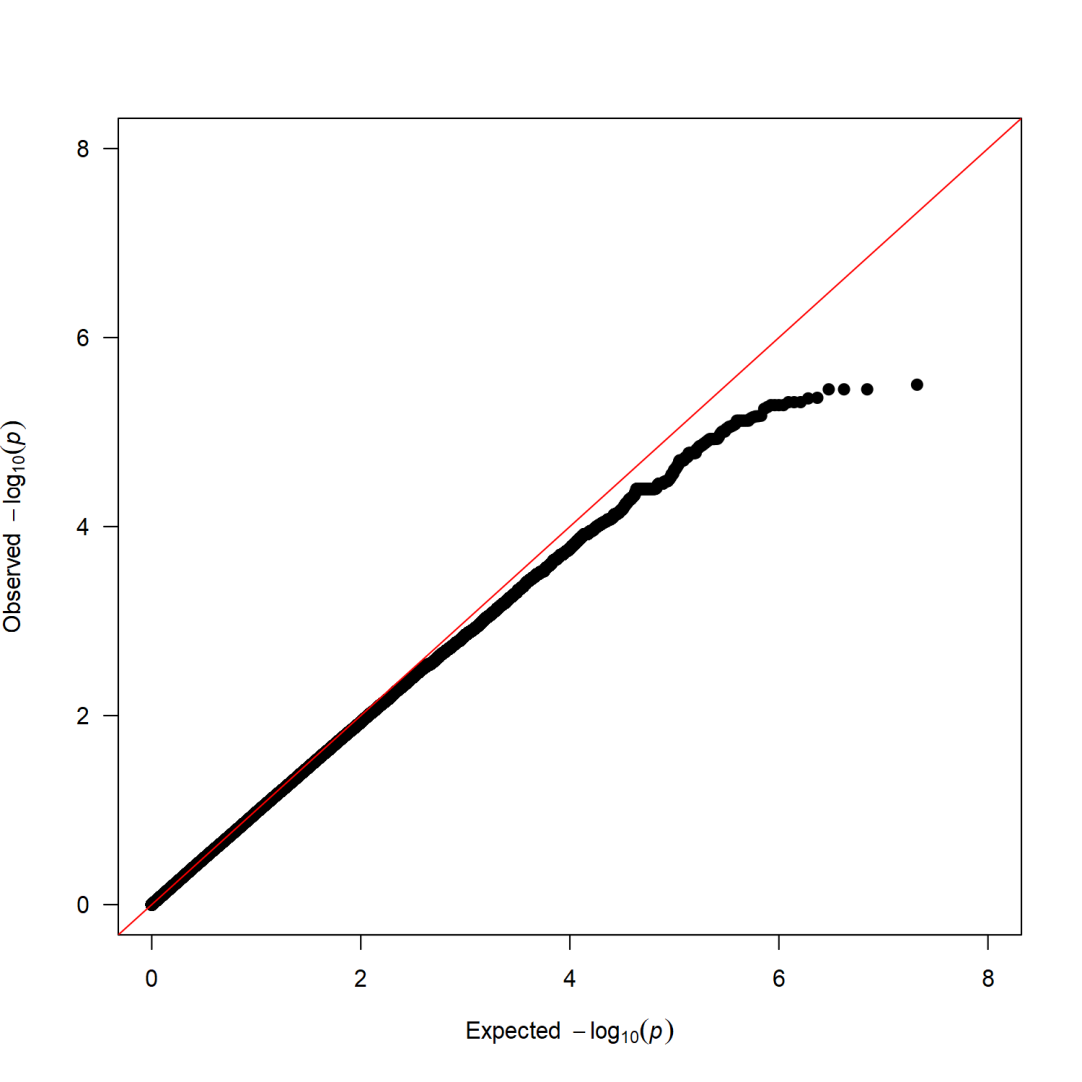


**Figure S17** Manhattan plot and QQ plot for methnoal
